# Anti-Inflammatory Cembrane-Type Diterpenoids and Prostaglandins from Soft Coral *Lobophytum sarcophytoides*

**DOI:** 10.3390/md17080481

**Published:** 2019-08-19

**Authors:** Hongjie Shen, Xiaowan Liu, Minghua Jiang, Guangyuan Luo, Zhenger Wu, Bin Chen, Jing Li, Lan Liu, Senhua Chen

**Affiliations:** 1 School of Marine Sciences, Sun Yat-sen University, Guangzhou 510006, China; 2 Shenzhen Key Laboratory for the Sustainable Use of Marine Biodiversity, Research Centre for the Oceans and Human Health, City University of Hong Kong Shenzhen Research Institute, Shenzhen 518057, China; 3 Southern Laboratory of Ocean Science and Engineering (Guangdong, Zhuhai), Zhuhai 519000, China

**Keywords:** soft coral, cembrane-type diterpenoid, prostaglandin, anti-inflammatory

## Abstract

Two new cembrane-type diterpenoids, lobophytins A (**1**) and B (**3**), and four new prostaglandins, (5*E*)-PGB_2_ (**10**), (5*E*)-13,14-dihydro-PGB_2_ (**11**), 13,14-dihydro-PGB_2_ (**12**) and 13,14-dihydro-PGB_2_-Me (**13**), together with ten known compounds were isolated from the soft coral *Lobophytum sarcophytoides*. The structures of these new secondary metabolites were identified by high resolution mass spectrometry (HR-ESIMS), nuclear magnetic resonance (NMR) and electron circular dichroism (ECD) analyses, as well as the modified Mosher’s method. Compounds **6**, **7**, **9**, **10**, **12**, **13**, **15** and **16** showed potential anti-inflammatory activity by inhibiting the production of nitric oxide (NO) in RAW264.7 cells that were activated by lipopolysaccharide, with IC_50_ values ranging from 7.1 to 32.1 μM and were better than the positive control indomethacin, IC_50_ = 39.8 μM.

## 1. Introduction

Cembrane-type diterpenoids are a large and structurally varied group of natural products derived from both terrestrial and marine organisms [1,2]. Among them, highly functionalized cembranoid diterpenes, containing a five-membered lactone moiety, have been mainly isolated from marine soft corals, especially from the genera *Lobophytum*, *Sarcophyton* and *Sinularia* [3,4]. Some of them have many kinds of pharmacological activities, such as cytotoxic [2,5], anti-inflammatory [6,7], anti-protozoal [8], antifouling [9] and anti-HIV [10].

Prostaglandins are biologically active lipid compounds, being 20-carbon fatty acids derived enzymically from the essential fatty acids by cyclization and oxidation [11,12]. They are widely distributed in animals and human-beings, which regulate a wide range of physiological activities. 

**Figure 1:**
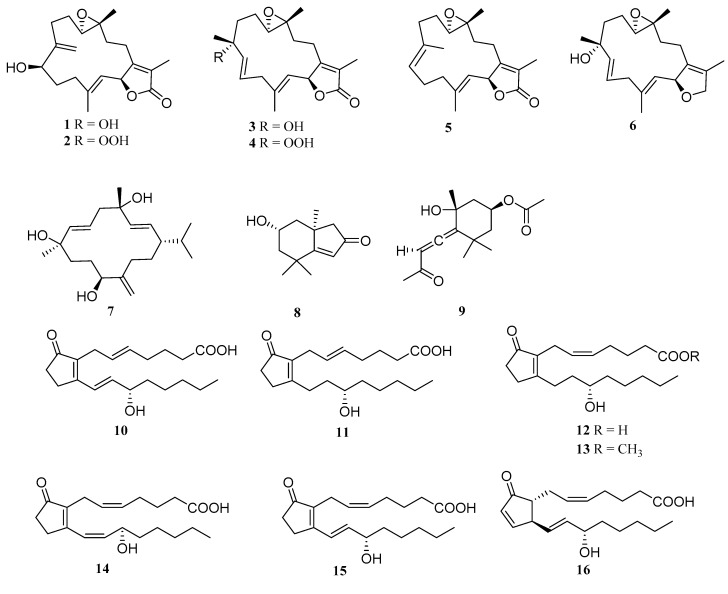
Chemical structures of **1**–**16**.

Recently, we searched for anti-inflammatory secondary metabolites from the South China Sea and the soft coral *Lobophytum sarcophytoides* was collected from Xisha Islands, China. EtOAc extract of soft coral *L. sarcophytoides* showed anti-inflammatory activity in vitro by inhibiting nitric oxide (NO) production in lipopolysaccharide activated in RAW264.7 cells. Subsequent chemical investigation led to the isolation of 16 secondary metabolites, including two new cembrane-type diterpenoids, lobophytins A (**1**) and B (**3**), and four new prostaglandins, (5*E*)-PGB_2_ (**10**), (5*E*)-13,14-dihydro-PGB_2_ (**11**), 13,14-dihydro-PGB_2_ (**12**) and 13,14-dihydro-PGB_2_-Me (**13**), as well as ten known compounds, sarcomililatin B (**2**), sarcomililatin A (**4**), (+)-isosarcophine (**5**), (1*S*,2*E*,4*S*,6*E*,8*S*,11*R*)-2,6,12(20)-cembratriene-4,8,11-triol (**7**), loliolide (**8**), *apo*-9′-fucoxanthinone (**9**), (13*E*)-PGB_2_ (**14**), PGB_2_ (**15**) and PGA_2_ (**16**) (Figure 1). Compounds **6**, **7**, **9**, **10**, **12**, **13**, **15** and **16** showed potential anti-inflammatory activity with IC_50_ values ranging from 7.1 to 32.1 μM. Herein, we report the isolation, structure determination and anti-inflammatory bioactivity of the secondary metabolites.

## 2. Results and Discussion

Compound **1** was obtained as colorless oil and its molecular formula was established as C_20_H_28_O_4_ (seven degrees of unsaturation) according to the negative HR-ESIMS ion at *m*/*z* 331.1906 [M − H]^−^ (calculated for C_20_ H_27_ O_4_, 331.1909) (Figure S1). The IR spectrum of **1** revealed the presence of hydroxy (3450 cm^−1^) group and an α, β-unsaturated *γ*-lactone moiety (1737 and 1675 cm^−1^) (Figure S2). The ^1^H NMR data (Table 1) showed resonances for two vinyl methyls [δ_H_ 1.79 (3H, s); 1.91 (3H, s)], a tertiary methyl [δ_H_ 1.21 (3H, s)], three olefinic protons [δ_H_ 4.99 (1H, dd, *J* = 10.0, 1.0 Hz); 4.92 (1H, d, *J* = 0.9 Hz); 5.19 (1H, t, *J* = 1.6 Hz)], three oxygenated methines [δ_H_ 5.64 (1H, dd, *J* = 10.0, 1.7 Hz); 3.83 (1H, dd, *J* = 10.7, 6.3 Hz); 2.69 (1H, dd, *J* = 10.3, 2.6 Hz)] (Figure S3). The ^13^C NMR data (Table 1) revealed the presence of 20 carbons belonging to three methyls, seven methylenes, four methines and six quaternary carbons. Detailed analysis of the ^1^H and ^13^C NMR data (Figure S4) suggested that **1** belongs to the cembrane-type diterpenoid class, which was quite similar to sarcomililatin A (**2**) [13].

**Figure 2:**
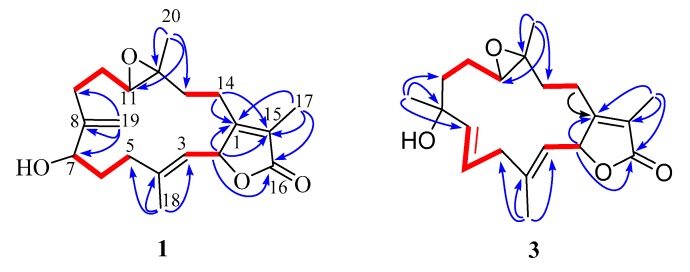
Key ^1^H-^1^H COSY (red line) and HMBC (blue arrow) correlations of compounds **1** and **3**.

**Figure 3:**
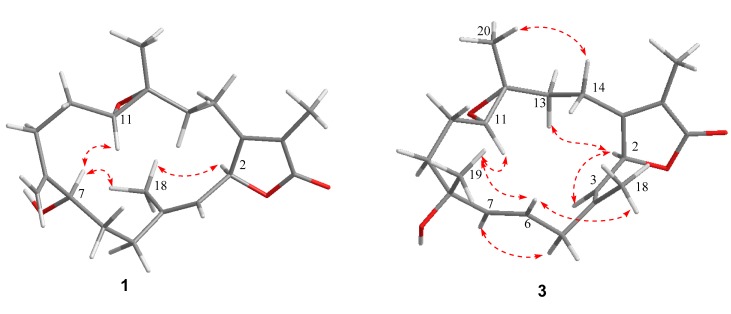
Key NOESY (red line) correlations of compounds **1** and **3**.

**Figure 4:**
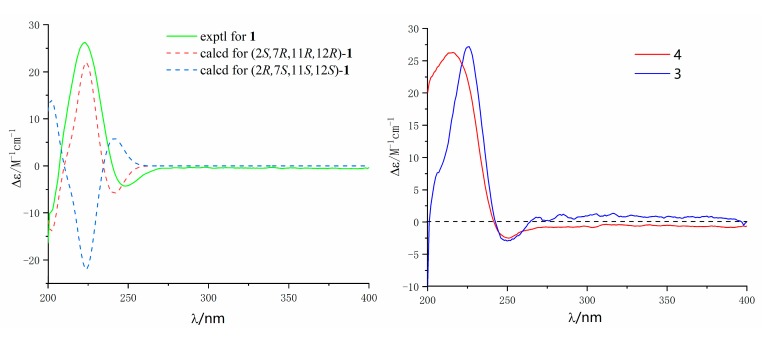
Experimental and calculated ECD spectra of compounds **1**, **3** and **4** (in MeOH).

The ^1^H-^1^H COSY spectrum (Figure S5) revealed the appearance of four isolated proton spin systems as depicted in Figure 2. The key heteronuclear multiple bond correlation (HMBC) correlations from H-17 to C-1, C-15 and C-16; H-18 to C-3, C-4 and C-5; H-19 to C-7, C-8 and C-9; H-20 to C-11, C-12 and C-13; H-2 to C-1, C-15 and C-16; and H-14 to C-1 and C-15 established the cembrane-type diterpenoid skeleton as shown in Figure 2. The epoxy ring was located at C-11 and C-12 on the base of the HMBC correlations from H-20 to C-11 and C-12, as well as the chemical shift values of C-11 (δ_C_ 62.1) and C-12 (δ_C_ 61.1). The position of the remaining hydroxy group was assigned at C-7 (δ_C_ 70.1) according to the HMBC correlations from H-19 to C-7 and the ^1^H-^1^H COSY correlation between H-6 and H-7. The relative configuration of **1** was deduced by analysis of the nuclear overhauser effect spectroscopy (NOESY) data (Figure 3). The observed NOE correlations H-18 with H-2 and H-7 indicated they are α-orientation. NOE correlations H-11 with H-7, but not with H-20 suggested that H-11 and H-20 are β and α-orientation, respectively. The absolute configuration of **1** was determined by comparing experimental and calculated ECD spectra. The predicted ECD spectrum of (2*S*, 7*R*, 11*R*, 12*R*)-1 was in good agreement with that of the experimental one (Figure 4). Finally, the gross structure of **1** was assigned as shown and was given the trivial name lobophytin A.

Compound **3** was isolated as colorless oil. The molecular formula of **3** was determined as C_20_H_28_O_4_ on the base of the positive HR-ESIMS ion at *m*/*z* 333.2058 [M − H]^+^ (calculated for C_20_H_29_O_4_, 333.2060) (Figure S9). The IR spectrum of **3** revealed the presence of hydroxy (3450 cm^−1^) group and an α, β-unsaturated γ-lactone moiety (1737 and 1675 cm^−1^) (Figure S10). The ^1^H and ^13^C NMR data (Table 1) of **3** revealed its structure possessed great similarity to the known cembrane-type diterpenoid, sarcomililatin B (**4**) [13]. The main difference between them was that the hydroperoxy group in **4** was replaced by the hydroxyl group in **3**. This replacement caused the chemical shift of C-8 to be shifted upfield Δδ_C_ 11.4 ppm (δ_C_ 72.6 in **3**; 84.0 in **4**). The significant ^1^H-^1^H COSY (Figure S13) and HMBC correlations (Figure 2) allowed the complete assignment for the planar structure of **3**. The relative configuration of **3** was deduced by analysis of the NOESY data (Figure 3). The observed NOE correlations H-2 with H-13a and H-3; H-5a with H-13a and H-3 indicated they are α-orientation. NOE correlations H-6 with H-18 and H-19; H-19 with H-11; H-20 with H-14a suggested that H-6, H-14a, H-11, H-18, H-19 and H-20 are β-orientation. Compound **3** and sarcomililatin B (**4**) [13] showed quite similar ECD spectra (Figure 4), with a positive Cotton effect at 223 nm (Δε = +27.2) and a negative Cotton effect at 248 nm (Δε = −2.9), indicating that they have the same absolute configurations as 2*S*, 8*R*, 11*R* and 12*R*. Therefore, the structure of **3** was elucidated as depicted, named as lobophytin B. 

**Figure 5:**
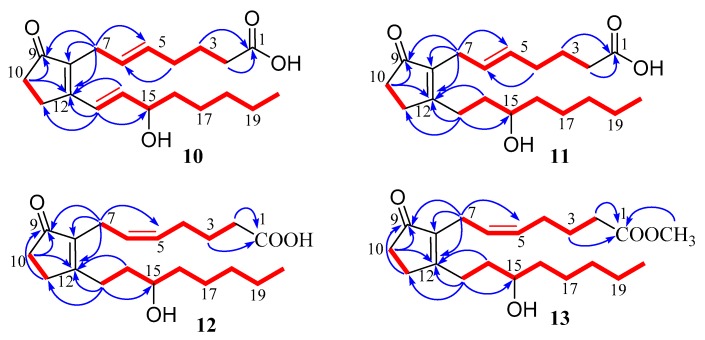
Key ^1^H-^1^H COSY (red line) and HMBC (blue arrow) correlations of compounds **10**–**13**.

Compound **10** was isolated as colorless oil. It has the molecular formula C_20_H_28_O_4_ (six degrees of unsaturation), as deduced from the negative HR-ESIMS ion at *m*/*z* 333.2068 [M − H]^−^ (calculated for C_20_H_29_O_4_, 333.2071) (Figure S17). The ^1^H and ^13^C NMR spectra (Figures S19 and S20) of compound **10** were similar to those of known prostaglandin, PGB_2_ (**15**), except for chemical shift variation of two olefinic (δ_C_ 131.8 and 128.3 in **10**; 27.7 and 22.1 in **15**) and two methylenes (δ_C_ 32.9 and 26.7 in **10**; 27.7 and 22.1 in **15**) carbons in ^13^C NMR spectrum (Table 2). This suggested that compound **10** was a geometric isomer of compound **15** with the replacement of 5*E* geometry of the double bond by 5*Z* (Figure 5) on the base of the ^13^C NMR chemical shifts of allylic methylene carbons (*Z* alkenes, δ_C_ < 28 ppm; *E* alkenes, δ_C_ > 30 ppm) of alkenes [14]. Detailed analysis of the 2D NMR spectroscopic data, the structure of **10** was established as (5*E*)-PGB_2_. 

Compound **11** was isolated as colorless oil and its molecular formula was established as C_20_H_28_O_4_ (five degrees of unsaturation) according to the negative HR-ESIMS ion at *m*/*z* 335.2231 [M − H]^−^ (calculated for C_20_H_31_O_4_, 335.2227) (Figure S25). The ^1^H and ^13^C NMR spectra (Figures S27 and S28) of compound **11** were similar to those of prostaglandin, (5*E*)-PGB_2_ (**10**), except for the absence of two olefinic proton (δ_H_ 6.37 and 6.86; δ_C_ 124.3 and 143.7) and the presence of two additional methylenes (δ_H_ 2.53, 2.61; 1.58, 1.67). It means that compound **11** was an analogue of (5*E*)-PGB_2_ (**10**) with the replacement of two olefinic by two methylene group, which was supported by the ^1^H-^1^H COSY correlations of H-14 with H-13 and H-15 (Figure 5). Detailed analysis of the 2D NMR spectroscopic data (Figures S29–S31), the structure of **11** was established as shown in Figure 2 and named as (5*E*)-13,14-dihydro-PGB_2_.

Compound **12** was isolated as colorless oil. The molecular formula of **12** was established as C_20_H_28_O_4_ (five degrees of unsaturation) according to the negative HR-ESIMS ion at *m*/*z* 335.2231 [M − H]^−^ (calculated for C_20_H_31_O_4_, 335.2227) (Figure S32). The ^1^H and ^13^C NMR spectra (Figures S34 and S35) of compound **12** were similar to those of (5*E*)-13,14-dihydro-PGB_2_ (**11**), except for chemical shift variation of two olefinic and two methylene carbons in ^13^C NMR spectrum (Table 2). This suggested that compound **12** was a geometric isomer of compound **11** with the replacement of 5*Z* geometry of the double bond by 5*E* according to the ^13^C NMR chemical shifts of allylic methylene carbons (*Z* alkenes, δ_C_ < 28 ppm; *E* alkenes, δ_C_ > 30 ppm) of alkenes. Therefore, the structure of **12** was established as 13,14-dihydro-PGB_2_.

Compound **13** was isolated as colorless oil. The molecular formula of **13** was assigned as C_20_H_28_O_4_ (five degrees of unsaturation) on the base of the negative HR-ESIMS ion at *m*/*z* 351.2535 [M − H]^−^ (calculated for C_21_H_35_O_4_, 351.2535) (Figure S40). The ^1^H and ^13^C NMR spectra (Figures S42 and S43) of compound **13** were quite similar to those of 13,14-dihydro-PGB_2_ (**12**), except for the presence of an additional methyl group at δ_H_ 3.62 (δ_C_ 51.5) (Table 2). The difference revealed that compound **13** was a methylated analogue of compound **12,** which was supported by the HMBC correlations from −OCH_3_ to C-1 (Figure 5). Finally, 2D NMR spectroscopic data (Figures S44–S46) further elucidated the structure of **13**, as shown in Figure 5 and named as 13,14-dihydro-PGB_2_-Me.

**Figure 6:**
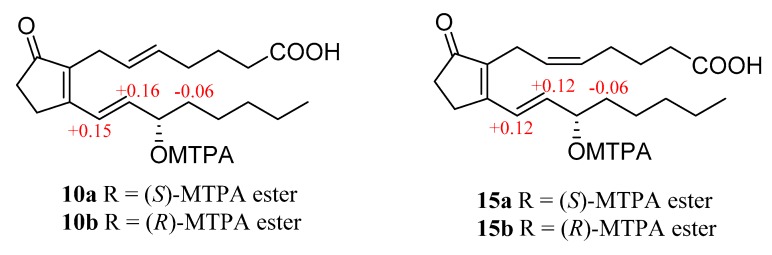
∆δ = δ_S_ − δ_R_ values in ppm obtained from the MTPA esters of **10** and **15**.

All isolated prostaglandins **10**–**16** shared a secondary alcohol at C-15. Their absolute configurations at C-15 were assigned by the modified Mosher’s method and comparison of optical activity sign, in combination with biosynthetic considerations. Initially, the known compound with most amount, PGB_2_ (**15**) was used to determine absolute configuration by application of the modified Mosher’s method because the chemical shifts of H-13 and H-14 at the double bond near the secondary alcohol at C-15 would change obviously after the reaction with the Mosher’s reagent. The reaction of **15** with (*R*)- and (*S*)-MTPA chloride afforded the corresponding (*S*)-MTPA ester (**15a**) and (*R*)-MTPA ester (**15b**). The chemical shifts for H-14 and H-16 of **15a** were δ_H_ 6.42 and 1.85, respectively, compared to δ_H_ 6.30 and 1.91 in **15b** (Figures S49 and S51). The chemical shift values (Δ*δ* = δ_S_ − δ_R_) (Figure 6) suggested that the absolute configuration of C-15 is *S*. Similarly, the absolute configuration of (5*E*)-PGB_2_ (**10**) was clearly resolved by the modified Mosher’s method and was characterized as *S*. Then, we also tried to use the modified Mosher’s method to analyse the absolute configuration of (5*E*)-13,14-dihydro-PGB_2_ (**11**), 13,14-dihydro-PGB_2_ (**12**) and 13,14-dihydro-PGB_2_-Me (**13**), but the chemical shifts of H-14 and H-16 are overlapped and difficult to classify with the help of heteronuclear singular quantum correlation (HSQC) and ^1^H-^1^H COSY. Finally, optical rotations of all isolated prostaglandins **10**–**16** were measured and they have the same optical activity sign. Thus, the configuration at the hydroxy-bearing C-15 was deduced as *S*, as well as in combination with biosynthetic considerations.

The known compound, sarcomililatin B (**2**) [13], sarcomililatin A (**4**) [13], (+)-isosarcophine (**5**) [15], (1*S*,2*E*,4*S*,6*E*,8*S*,11*R*)-2,6,12(20)-cembratriene-4,8,11-triol (**7**) [16], loliolide (**8**) [17], *apo*-9′-fucoxanthinone (**9**) [18], (13*Z*)-PGB_2_ (**14**) [19], PGB_2_ (**15**) [19,20] and PGA_2_ (**16**) [19] were identified by NMR, ESI-MS and optical rotation data analysis and comparison of spectroscopic data with literatures.

All isolated compounds (except for **14** with limited amount) were tested for their inhibition activity against LPS-activated NO production in RAW264.7 cells using the Griess assay. Two cembrane-type diterpenoids **6** and **7** displayed promising inhibitory effects on the production of NO with IC_50_ values 26.7 and 17.6 μM (the positive control indomethacin, IC_50_ = 39.8 μM). apo-9′-Fucoxanthinone (**9**) had good inhibition activity against LPS-activated NO production in RAW264.7 cells with 32.1 μM. Most of the isolated prostaglandins, **10**, **12**, **13**, **15** and **16** showed potential anti-inflammatory activity with IC_50_ values 20.4, 24.8, 16.1, 15.9 and 7.1 μM, respectively. The other compounds were displayed weak or not anti-inflammatory. To evaluate the effects of all tested compounds on cell proliferation/viability, none of the compounds (up to 50 μM) showed any significant cytotoxicity with LPS treatment for 24 h using the thiazolyl blue tetrazolium bromide (MTT) method. The structure−activity relationships revealed that a methylene group at C-16 of five-member ring among cembrane-type diterpenoids was much more favorable than a carbonyl group for activity, as compound **5** was much more active than compounds **1**−**4**. Among prostaglandins with 5*E* geometry, double bond at C-13 played an important role in their anti-inflammatory action because (5*E*)-PGB_2_ (**10**) was much more active than (5*E*)-13,14-dihydro-PGB_2_ (**11**). The side chain possessing a terminal carbonyl acid group made a more positive contribution to the anti-inflammatory activity than those with methoxycarbonyl group (**12** vs. **13**). The structure of PGA_2_ made a more positive contribution to the anti-inflammatory activity than those of PGB_2_ (**16** vs. **15**). In addition, the secondary metabolites (except for **14** with limited amount) were evaluated for their cytotoxicity using A549 (lung cancer), HepG2 (liver cancer) and MCF-7 (breast cancer) human cell lines and showed no cytotoxicity against all three cell lines at 50 μM.

## 3. Materials and Methods

### 3.1. General Experimental Procedures

Optical rotations were recorded on an MCP 200 (Anton Paar, Shanghai, China) polarimeter. UV data were measured on a Shimadzu UV-240 spectrophotometer (Shimadzu, Kyoto, Japan). IR spectra were detected on a Fourier transformation infra-red spectrometer coupled with infra-red microscope EQUINOX 55 (Bruker, Rheinstetten, Germany). 1D and 2D NMR spectra were performed on a Bruker Avance 400 MHz spectrometer with tetramethylsilane as internal standard. HR-ESIMS data were carried out on an LTQ-Orbitrap LC-MS spectrometer (Thermo Corporation, Waltham, MA, USA). ESIMS spectra were obtained on an ACQUITY QDA (Waters Corporation, Milford, MA, USA). HPLC was carried out on Essentia LC-16 with an SPD-16 Detector (Shimadzu, Shanghai, China). Column chromatography was performed on silica gel (200–300 mesh, Qingdao Marine Chemical Factory, China) and Sephadex LH-20 (GE Healthcare, Littile Chalfont, UK).

### 3.2. Biological Material

*Lobophytum sarcophytoides* SYSU-MS001 were collected along the coast of Xisha Islands (17°06′14.50″ N, 111°28′35.03″ E), South China Sea, in July 2018, at a depth of −25 m and were frozen immediately after collection. The soft coral has been preserved at the school of marine sciences, Sun Yat-Sen University.

### 3.3. Extraction, Isolation and Characterization

The soft coral (0.9 kg) was cut into small pieces and extracted three times with CH_2_Cl_2_/MeOH (1:1, 1 L) to afford the organic extract (10.3 g). The extract was subjected to silica gel column eluted with PE-EtOAc (from 80:20 to 0:100) to give seven fractions (A–F). Fr. B was separated by Sephadex LH-20 (CC, 3 × 50 cm) eluted with MeOH-CH_2_Cl_2_ (1:1) to give Fr.B.1-Fr.B.4. Fr.B.2 was further subjected to silica gel CC by elution by PE-EtOAc (75:25) to afford Fr.B.2.1-Fr.B.2.6 and Fr.B.2.3 was compound 5 (5 mg). Fr.B.2.4 was separated by the PR-HPLC (MeOH- H_2_O 75:25, flow rate 1.5 mL/min, Ultimate C_18_ column 10 × 250 nm, 5 μm) to give **4** (3 mg, t_R_ = 15.5 min) and **2** (2 mg, t_R_ = 17.0 min). Fr.B.3 was subjected to silica gel CC by elution by PE-EtOAc (75:25) to afford Fr.B.3.1- Fr.B.3.6, and Fr.B.3.5 was compound **6** (6 mg). Then Fr.B.3.5 was applied to the normal-phase HPLC (n-hexane/2-propanol 92:8, flow rate 1.5 mL/min, Ultimate SiO_2_ column 10 × 250 nm, 5 μm) to afford **9** (5 mg, t_R_ = 19.0 min), **3** (2 mg, t_R_ = 21.5 min). Fr. C was applied to a Sephadex LH-20 column with MeOH-CH_2_Cl_2_ (1:1) to Fr.C.2.1-Fr.C.2.5 and Fr.C.2 was fractionated on a silica gel column with PE-EtOAc (70:30) to give five fractions (Fr.C.2.1-Fr.C.2.5). Fr.C.2.3 was purified by the normal-phase HPLC (n-hexane/ 2-propanol 90:10, flow rate 1.5 mL/min, Ultimate SiO_2_ column 10 × 250 nm, 5 μm) to give **1** (3 mg, t_R_ = 23 min) and **10** (8 mg, t_R_ = 26 min). Fr.C.2.4 was separated by PR-HPLC (MeOH-H_2_O 75:25, flow rate 1.5 mL/min, ACE 5 C18-PFP column 250 × 10 mm, 5 μm) to give **8** (4 mg, t_R_ = 17 min). Fr. D was subjected to a Sephadex LH-20 column with MeOH-CH_2_Cl_2_ (1:1) to Fr.D.4.1- Fr.D.4.4, then Fr.D.4.4 was purified by PR-HPLC (MeOH-H_2_O 75:25, flow rate 1.5 mL/min, ACE 5 C18-AR column 250 × 10 mm, 5 μm) to obtain **16** (10 mg, t_R_ = 16.5 min), **15** (25 mg, t_R_ = 24 min) and **11** (12 mg, t_R_ = 18.5 min). Fr. E was subjected to a Sephadex LH-20 column with MeOH-CH_2_Cl_2_ (1:1) to Fr.E.1-Fr.E.4, then Fr.E.3 was fractionated on a silica gel column with PE-EtOAc (70:30) to give four fractions (Fr.E.3.1- Fr.E.3.4). Fr.E.3.3 was purified by PR-HPLC (MeOH-H_2_O 75:25, flow rate 1.5 mL/min, ACE 5 C18-AR column 250 × 10 mm, 5 μm) to obtain **12** (5 mg, t_R_ = 17 min), **13** (7 mg, t_R_ = 19 min) and **14** (1 mg, t_R_ = 22 min). Fr.E.3.4 was also purified by PR-HPLC (MeOH-H_2_O 75:25, flow rate 1.5 mL/min, ACE 5 C18-AR column 250 × 10 mm, 5 μm) to give **7** (5 mg, t_R_ = 15 min).

#### 3.3.1. Lobophytin A (**1**)

Colorless oil; ${\left[\alpha \right]}_{D}^{20}$ = 53.1 (MeOH, *c* 0.05); UV (MeOH) *λ*_max_ (log *ε*) 212 (4.12) nm; CD (MeOH) *λ*_max_ (Δε) 223 (+26.3), 248 (−2.5) nm; IR (neat) *ν*_max_ 3450, 2921, 2854, 1737, 1675, 1446, 1093, 998, 904 cm^−1^; ^1^H NMR (400 MHz, MeOD) and ^13^C NMR (100 MHz, MeOD) data see Table 1; HR-ESIMS *m*/*z* 331.1906 [M − H]^−^ (calculated for C_20_ H_27_ O_4_, 331.1909).

#### 3.3.2. Lobophytin B (**3**)

Colorless oil; ${\left[\alpha \right]}_{D}^{20}$ = 33.3 (MeOH, *c* 0.06); UV (MeOH) *λ*_max_ (log *ε*) 209 (4.10), 280 (3.74) nm; CD (MeOH) *λ*_max_ (Δ*ε*) 216 (+27.2), 250 (−2.9) nm; IR (neat) *ν*_max_ 3422, 2922, 2853, 1733, 1671, 1438, 1364, 1243, 981 cm^−1^; ^1^H NMR (400 MHz, MeOD) and ^13^C NMR (100 MHz, MeOD) data see Table 1; HR-ESIMS *m*/*z* 333.2058 [M − H]^+^ (calculated for C_20_H_29_O_4_, 333.2060).

#### 3.3.3. (5*E*)-PGB_2_ (**10**)

Colorless oil; ${\left[\alpha \right]}_{D}^{20}$ 1.3 (c 0.21, MeOH); UV (MeOH) *λ*_max_ (log *ε*) 280 (4.06); IR (neat) *ν*_max_ 3357, 3191, 2920, 2852, 1694, 1624, 1415 cm^−1^; ^1^H NMR (400 MHz, MeOD) and ^13^C NMR (100 MHz, MeOD) data see Table 2; HR-ESIMS *m/z* 333.2068 [M − H]^−^ (calculated for C_20_H_29_O_4_, 333.2071).

#### 3.3.4. (5*E*)-13,14-dihydro-PGB_2_ (**11**)

Colorless oil; ${\left[\alpha \right]}_{D}^{20}$ 0.4 (c 0.13, MeOH); UV (MeOH) *λ*_max_ (log ε) 237 (4.07); IR (neat) *v*_max_ 3392, 2939, 2858, 1691, 1637, 1454, 1363, 1250, 1055 cm^−1^; ^1^H NMR (400 MHz, MeOD) and ^13^C NMR (100 MHz, MeOD) see Table 2; HR-ESIMS *m*/*z* 335.22312 [M − H]^−^ (calculated for C_20_H_31_O_4_, 335.22278).

#### 3.3.5. 13,14-dihydro-PGB_2_ (**12**)

Colorless oil; ${\left[\alpha \right]}_{D}^{20}$ 0.3 (*c* 0.29, MeOH); UV (MeOH) λ_max_ (log *ε*) 238 (4.11); IR (neat) *v*_max_ 3413, 2935, 2868, 1697, 1628, 1450, 1358, 1250, 1240, 1051 cm^−1^; ^1^H NMR (400 MHz, MeOD) and ^13^C NMR (100 MHz, MeOD) see Table 2; HR-ESIMS *m/z* 335.22312 [M − H]^−^ (calculated for C_20_H_31_O_4_, 335.22278).

#### 3.3.6. 13,14-dihydro-PGB_2_-Me (**13**)

Colorless oil; ${\left[\alpha \right]}_{D}^{20}$ 0.9 (*c* 0.20, MeOH); UV (MeOH) *λ*_max_ (log *ε*) 237 (4.09); IR (neat) ν_max_ 3445, 2931, 2858, 1737, 1695, 1635, 1440, 1361, 1045 cm^−1^; ^1^H NMR (400 MHz, MeOD) and ^13^C NMR (100 MHz, MeOD) see Table 2; HR-ESIMS *m/z* 351.2535 [M − H]^−^ (calculated for C_21_H_35_O_4_, 351.2535).

### 3.4. Preparation of (S)-MTPA Ester and (R)-MTPA Ester

#### 3.4.1. (*S*)-MTPA Ester (**15a**) and (*R*)-MTPA Ester (**15b**)

A sample of **15** (1.0 mg, 4 μmol), (*R*)-MPTACl (5.0 μL, 25 μmol) and pyridine-*d*_5_ (0.5 mL) were used to react in an NMR tube at room temperature for 24 h, with the ^1^H NMR data of the (*S*)-MTPA ester derivative (**15a**) were obtained directly on the reaction mixture [21,22]. ^1^H NMR (selected signals, pyridine-*d*_5_, 400 MHz) δ_H_: 7.21 (1H, d, H-13), 6.42 (1H, dd, H-14), 5.95 (1H, q, H-15), 1.85 (2H, m, H-16).

Similarly, the reaction mixture from another sample of **15** (1.0 mg, 4 μmol), (*S*)-MPTACl (5.0 μL, 26 μmol) and pyridine-*d*_5_ (0.5 mL) was processed as described above for **15a** to afford **15b**. ^1^H NMR (selected signals, pyridine-*d*_5_, 400 MHz) *δ*_H_: 7.10 (1H, d, H-13), 6.30 (1H, dd, H-14), 5.94 (1H, q, H-15), 1.91 (2H, m, H-16).

#### 3.4.2. (*S*)-MTPA Ester (**10a**) and (*R*)-MTPA Ester (**10b**)

Similar method was allowed to afford (*S*)-MTPA ester (**10a**) and (*R*)-MTPA Ester (**10b**). ^1^H NMR (selected signals, pyridine-*d*_5_, 400 MHz) **10a** *δ*_H_: 7.26 (1H, d, H-13), 6.46 (1H, dd, H-14), 5.95 (1H, q, H-15), 1.85 (2H, m, H-16). **10b** *δ*_H_: 7.11 (1H, d, H-13), 6.30 (1H, dd, H-14), 5.93 (1H, q, H-15), 1.91 (2H, m, H-16).

### 3.5. Calculation of ECD Spectra

Molecular Merck force field (MMFF) and density functional theory/time-dependent density functional theory (DFT/TD-DFT) calculations were performed on Spartan’14 (Wavefunction, Inc., Irvine, CA, USA) and the Gaussian 09 software (Gaussian, Wallingford, CT, USA), respectively [23]. Using DFT calculations at the B3LYP/6-31G (d) level was to generate and optimize conformers. Conformers with a Bolzmann distribution over 1% were chosen for ECD calculations in MeOH at the B3LYP/6311+G (2d, p) level. ECD spectra were generated by the software SpecDis 1.7.1 (University of Würzburg, Würzburg, Germany) and Origin Pro 8.5 (OriginLab, Ltd., Northampton, MA, USA) from dipole-length rotational strengths by applying Gaussian band shapes with sigma = 0.16 eV. All calculations were carried out on Tianhe-2 in the National Super Computer Center in Guangzhou.

### 3.6. Cytotoxic Assay

A549 (lung cancer), HepG2 (liver cancer) and MCF-7 (breast cancer) was used to evaluate the cytotoxicity of all tested compounds. The three human tumor cell lines human lung adenocarcinoma (A549), human hepatocellular carcinoma (HepG2), and the human breast adenocarcinoma cell line (MCF-7) were bought from the cell bank of the Chinese Academy of Sciences (Shanghai, China). The cytotoxic activities of the tested compounds were assayed according to the MTT method by using 96 well plates on the base of the previous reported procedures [24].

### 3.7. Anti-Inflammation Bioassays

The anti-inflammation activity of the isolated compounds was evaluated according to the reported procedures [25].

## 4. Conclusions

In summary, two new cembrane-type diterpenoids, lobophytins A (**1**) and B (**3**), and four new prostaglandins, (5*E*)-PGB_2_ (**10**), (5*E*)-13,14-dihydro-PGB_2_ (**11**), 13,14-dihydro-PGB_2_ (**12**) and 13,14-dihydro-PGB_2_-Me (**13**), together with ten known compounds were isolated from the soft coral *Lobophytum sarcophytoides*. The structures of new compounds were determined by analysis of HR-ESIMS, 1D and 2D NMR spectroscopic data and the absolute configurations were further determined by comparison of the experimental and calculated ECD spectra, as well as the modified Mosher’s method. Two cembrane-type diterpenoid displayed promising inhibitory effects on the production of NO with IC_50_ values 26.7 and 17.6 μM (the positive control indomethacin, IC_50_ = 39.8 μM). Most of the isolated prostaglandins, **10**, **12**, **13**, **15** and **16** showed potential anti-inflammatory activity with IC_50_ values ranging from 7.1 to 24.8 μM.

## Figures and Tables

**Table 1:** ^1^H (400 MHz) and ^13^C (100 MHz) NMR data of compounds **1** and **3** (in MeOH-*d*_4_, *J* in Hz).

No.	1		3	
	δ_C_	δ_H_, Mult.	δ_C_	δ_H_, Mult.
1	162.8, C		161.4, C	
2	79.8, CH	5.64, dd (10.0, 1.7)	79.2, CH	5.42, d (10.2)
3	122.9, CH	4.99, dt (10.0, 1.0)	120.1, CH	4.90, d (10.2)
4	143.5, C		143.7, C	
5	36.7, CH_2_	2.19, m 2.56, ddd (13.1, 9.9, 3.3)	41.7, CH_2_	2.73, dd (13.8, 7.2) 2.81, dd (13.8, 7.2)
6	32.9, CH_2_	1.40, m 2.00, m	125.2, CH	5.83, dt (15.6, 7.2)
7	70.1, CH	3.83, dd (10.7, 6.3)	140.2, CH	5.68, d (15.6)
8	155.1, C		72.6, C	
9	31.3, CH_2_	2.13, m 2.37, (13.7, 7.9, 2.8)	39.6, CH_2_	1.81, m
10	31.7, CH_2_	1.41, m	24.1, CH_2_	1.64, m 1.77, m
11	62.1, CH	2.69, dd (10.3, 2.6)	62.0, CH	2.76, m
12	61.1, C		60.1, C	
13	35.0, CH_2_	1.56, m 1.91, m	35.7, CH_2_	1.27, m 1.9, m
14	22.4, CH_2_	2.27, m	23.7, CH_2_	2.20, m 2.38, m
15	123.7, C		123.8, C	
16	174.5, C		174.7, C	
17	8.8, CH_3_	1.79, s	9.1, CH_3_	1.85, s
18	16.0, CH_3_	1.91, s	17.4, CH_3_	1.83, s
19	110.5, CH_2_	4.92, d (0.9) 5.19, t (1.6)	28.5, CH_3_	1.41, s
20	17.9, CH_3_	1.21, s	16.7, CH_3_	1.29, s

**Table 2:** ^1^H (400 MHz) and ^13^C (100 MHz) NMR data of compounds **10**–**13**.

No.	10 ^a^		11 ^a^		12 ^a^		13 ^b^	
	δ_C_, Type	δ_H_, Mult (J in Hz)	δ_C_, Type	δ_H_, Mult (J in Hz)	δ_C_, Type	δ_H_, Mult (J in Hz)	δ_C_, Type	δ_H_, Mult (J in Hz)
1	177.6, C		178.0, C		174.1, C		177.8, C	
2	34.7, CH_2_	2.19, t (6.9)	34.6, CH_2_	2.22, t (7.4)	33.8, CH_2_	2.33, t (7.5)	34.6, CH_2_	2.30, t (7.4)
3	26.0, CH_2_	1.59, m	25.9, CH_2_	1.61, m	25.6, CH_2_	1.66, m	26.1, CH_2_	1.66, m
4	32.9, CH_2_	1.97, m	32.9, CH_2_	1.99, m	27.2, CH_2_	2.20, q (7.2)	27.7, CH_2_	2.20, q (7.1)
5	131.8, CH	5.37, m	131.5, CH	5.39, m	129.8, CH	5.32, m	130.6, CH	5.34, m
6	128.3, CH	5.37, m	128.4, CH	5.39, m	128.2, CH	5.32, m	128.0, CH	5.30, m
7	26.7, CH_2_	2.95, m	26.8, CH_2_	2.89, m	21.9, CH_2_	2.92, m	22.1, CH_2_	2.94, d (6.6)
8	139.3, C		139.3, C		139.1, C		139.9, C	
9	211.9, C		212.4, C		208.3, C		212.3, C	
10	34.7, CH_2_	2.40, m	35.2, CH_2_	2.36, m	34.5, CH_2_	2.25, m	35.2, CH_2_	2.35, m
11	26.7, CH_2_	2.71, m	30.4, CH_2_	2.59, m	29.6, CH_2_	2.55, m	30.3, CH_2_	2.58, m
12	167.5, C		178.8, C		174.6, C		178.2, C	
13	124.3, CH	6.86, d (15.7)	28.7, CH_2_	2.53, m	28.3, CH_2_	2.55, m	28.8, CH_2_	2.55, m
				2.61, m		2.62, m		2.61, m
14	143.7, CH	6.37, dd (15.7, 5.8)	35.9, CH_2_	1.58, m	36.0, CH_2_	1.59, m	35.9, CH_2_	1.59, m
				1.67, m		1.69, m		1.67, m
15	72.8, CH	4.23, q (5.8)	72.2, CH	3.53	71.2, CH	3.57	72.2, CH	3.53, m
16	38.1, CH_2_	1.54, m	38.4, CH_2_	1.45, m	38.4, CH_2_	1.45, m	38.4, CH_2_	1.45, m
17	26.2, CH_2_	1.32, m	26.5, CH_2_	1.32, m	26.2, CH_2_	1.34, m	26.5, CH_2_	1.32, m
		1.41, m		1.45, m		1.46, m		1.45, m
18	32.9, CH_2_	1.31, m	33.1, CH_2_	1.31, m	32.7, CH_2_	1.30, m	33.1, CH_2_	1.31, m
19	23.7, CH_2_	1.31, m	23.7, CH_2_	1.32, m	23.4, CH_2_	1.31, m	23.7, CH_2_	1.32, m
20	14.4, CH_3_	0.88, t (6.7)	14.3, CH_3_	0.90, t (6.9)	14.3, CH_3_	0.88 t (6.7)	14.4, CH_3_	0.90, t (6.7)
OCH_3_							51.5, CH_3_	3.62, s

^a^ in MeOH-*d*_4_, ^b^ in acetone-*d*_6_.

## Supplementary Materials

The following are available online at https://www.mdpi.com/1660-3397/17/8/481/s1, Figures S1–S8: HR-ESIMS, IR, 1D and 2D NMR spectra of compound **1**; Figures S9–S16: HR-ESIMS, IR, 1D and 2D NMR spectra of compound **2**; Figures S17–S24: HR-ESIMS, IR, 1D and 2D NMR spectra of compound **10**; Figures S25–S31: HR-ESIMS, IR, 1D and 2D NMR spectra of compound **11**; Figures S32–S39: HR-ESIMS, IR, 1D and 2D NMR spectra of compound **12**; Figures S40–S46: HR-ESIMS, IR, 1D and 2D NMR spectra of compound **13**; Figures S47–S52: ^1^H NMR and ^1^H-^1^H COSY spectra of **10a**, **10b**, **15a** and **15b.**
