# Supplementary material for: Anti-Inflammatory Cembrane-Type Diterpenoids and Prostaglandins from Soft Coral Lobophytum sarcophytoides
Source: Mar Drugs. 2019 Aug 19;17(8):481. doi: 10.3390/md17080481 (PMC6723591; doi:10.3390/md17080481)

Supplementary materials for

Anti-inflammatory cembrane-type diterpenoids and prostaglandins from soft coral *Lobophytum sarcophytoides*

Hongjie Shen [1,‡], Xiaowan Liu [2,‡], Minghua Jiang [1], Guangyuan Luo [1], Zhenger Wu [1], Bin Chen [1], Jing Li [1], Lan Liu [1,3,*] , and Senhua Chen [1,3,*]

1 School of Marine Sciences, Sun Yat-sen University, Guangzhou 510006, People's Republic of China; shenhj5@mail2.susy.edu.cn; jiangmh23@mail2.sysu.edu.cn; luogy5@mail2.sysu.edu.cn; wuzher@mail2.sysu.edu.cn; chenbin25@mail.sysu.edu.cn; lijing356@mail.sysu.edu.cn

2 Shenzhen Key Laboratory for the Sustainable Use of Marine Biodiversity, Research Centre for the Oceans and Human Health, City University of Hong Kong Shenzhen Research Institute, Shenzhen 518057, China; xiaowl2222-x@my.cityu.edu.hk;

3 Southern Laboratory of Ocean Science and Engineering (Guangdong, Zhuhai), Zhuhai 519000, China;

* Correspondence: chensenh@mail.sysu.edu.cn; cesllan@mail.sysu.edu.cn. Tel.: +86-020-84725459;

‡ These authors contributed equally to this work.

Figure S1. HR-ESIMS of **1**. 4
Figure S2. IR spectrum of **1.** 4
Figure S3. ^{1}H (400 MHz) NMR spectrum of **1** in acetone-d_6. 5
Figure S4. ^{13}C NMR spectrum of **1** in acetone-d_6. 5
Figure S5. ^{1}H-^{1}H COSY spectrum of **1** in acetone-d_6. 6
Figure S6. HSQC spectrum of **1** in acetone-d_6. 6
Figure S7. HMBC spectrum of **1** in acetone-d_6. 7
Figure S8. NOESY spectrum of **1** in acetone-d_6. 8
Figure S9. HR-ESIMS of **3** 8
Figure S10. IR spectrum of **3** 9
Figure S11. ^{1}H (400 MHz) NMR spectrum of **3** in $CDCl_3$ 9
Figure S12. ^{13}C NMR spectrum of **3** in $CDCl_3$ 10
Figure S13. ^{1}H-^{1}H COSY spectrum of **3** in $CDCl_3$ 10
Figure S14. HSQC spectrum of **3** in $CDCl_3$ 11
Figure S15. HMBC spectrum of **3** in $CDCl_3$ 11
Figure S16. NOESY spectrum of **3** in $CDCl_3$. 12
Figure S17. HR-ESIMS of **10** 12
Figure S18. IR spectrum of **10** 13
Figure S19. ^{1}H (400 MHz) NMR spectrum of **10** in MeOH-d_4. 13
Figure S20. ^{13}C NMR spectrum of **10** in MeOH-d_4. 14
Figure S21. ^{1}H-^{1}H COSY spectrum of **10** in MeOH-d_4. 14
Figure S22. HSQC spectrum of **10** in MeOH-d_4. 15
Figure S23. HMBC spectrum of **10** in MeOH-d_4 16
Figure S24. NOESY spectrum of **10** in MeOH-d_4 16
Figure S25. HR-ESIMS of **11** 17
Figure S26. IR spectrum of **11** 17
Figure S27. ^{1}H (400 MHz) NMR spectrum of **11** in MeOH-d_4 18
Figure S28. ^{13}C NMR spectrum of **11** in MeOH-d_4 18
Figure S29. ^{1}H-^{1}H COSY spectrum of **11** in MeOH-d_4. 19
Figure S30. HSQC spectrum of **11** in MeOH-d_4. 19
Figure S31. HMBC spectrum of **11** in MeOH-d_4 20

Figure S32. HR-ESIMS of **12** 21
Figure S33. IR spectrum of **12** 21
Figure S34. ^{1}H (400 MHz) NMR spectrum of **12** in MeOH-d_4 22
Figure S35. ^{13}C NMR spectrum of **11** in MeOH-d_4 22
Figure S36. ^{1}H-^{1}H COSY spectrum of **12** in MeOH-d_4 23
Figure S37. HSQC spectrum of **12** in MeOH-d_4 23
Figure S38. HMBC spectrum of **12** in MeOH-d_4 24
Figure S39. NOESY spectrum of **12** in MeOH-d_4 24
Figure S40. HR-ESIMS of **13** 25
Figure S41. IR spectrum of **13** 25
Figure S42. ^{1}H (400 MHz) NMR spectrum of **13** in acetone-d_6. 26
Figure S43. ^{13}C NMR spectrum of **13** in acetone-d_6. 26
Figure S44. ^{1}H-^{1}H COSY spectrum of **13** in acetone-d_6. 27
Figure S45. HSQC spectrum of **13** in acetone-d_6. 27
Figure S46. HMBC spectrum of **13** in acetone-d_6 28
Figure S47. ^{1}H (400 MHz) NMR spectrum of **10a** in pyridine-d_5 28
Figure S48. ^{1}H (400 MHz) NMR spectrum of **10b** in pyridine-d_5 29
Figure S49. ^{1}H (400 MHz) NMR spectrum of **15a** in pyridine-d_5 29
Figure S50. ^{1}H-^{1}H COSY spectrum of **15a** in pyridine-d_5 30
Figure S51. ^{1}H (400 MHz) NMR spectrum of **15b** in pyridine-d_5 30
Figure S52. ^{1}H-^{1}H COSY spectrum of **15b** in pyridine-d_5 31

Figure S1. HR-ESIMS of **1**.

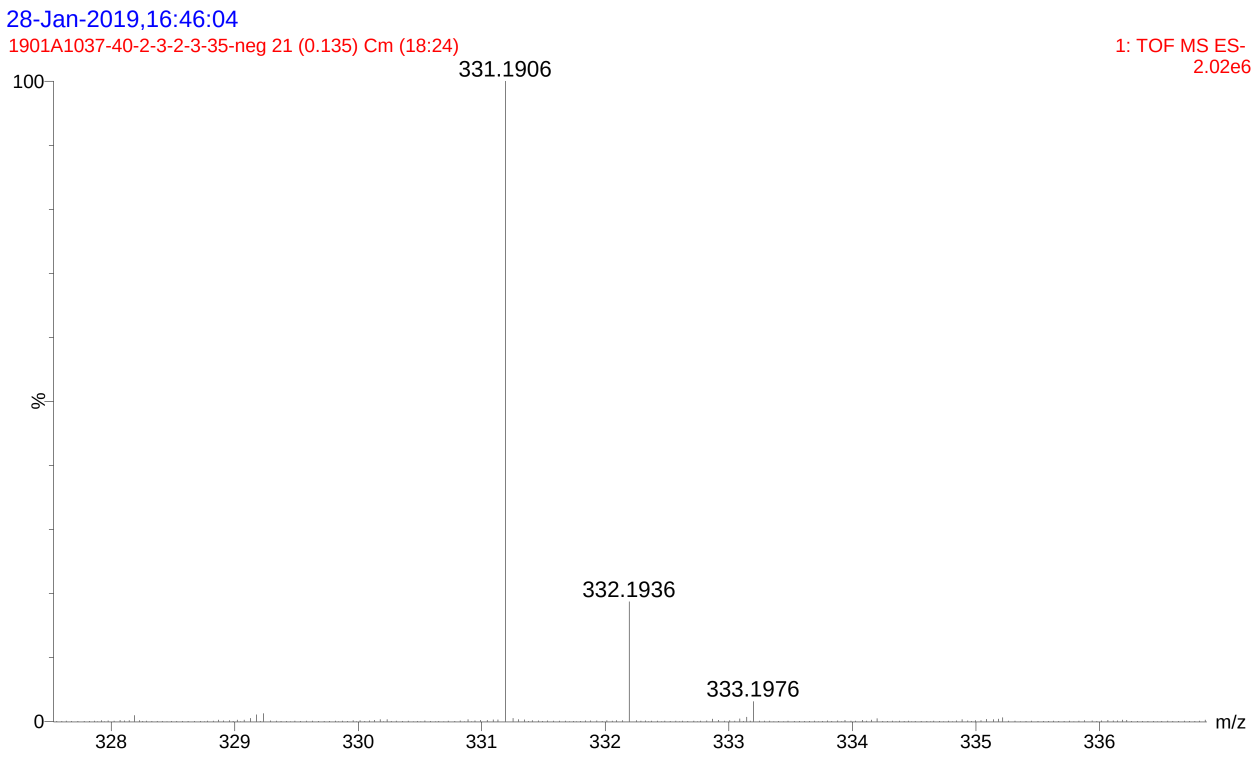

Figure S2. IR spectrum of **1.**

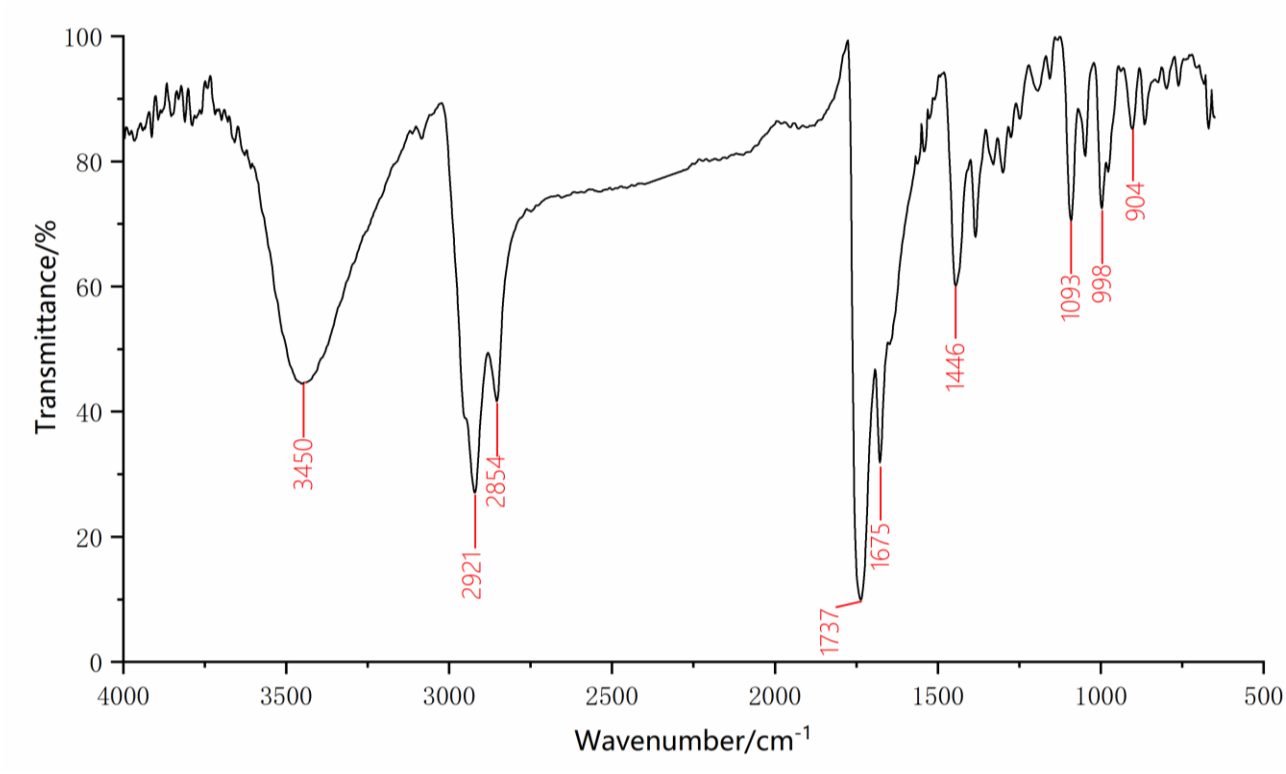

Figure S3. ^{1}H (400 MHz) NMR spectrum of **1** in acetone-*d*6.

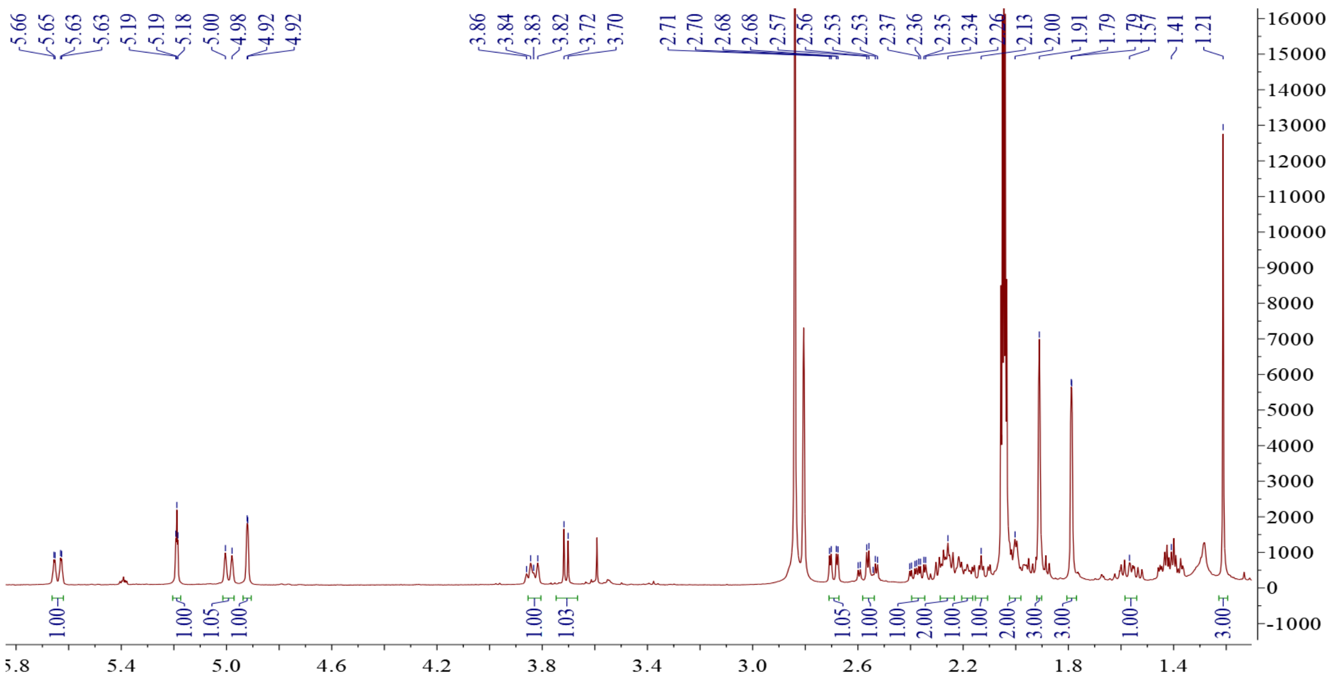

Figure S4. ^{13}C NMR spectrum of **1** in acetone-*d*6.

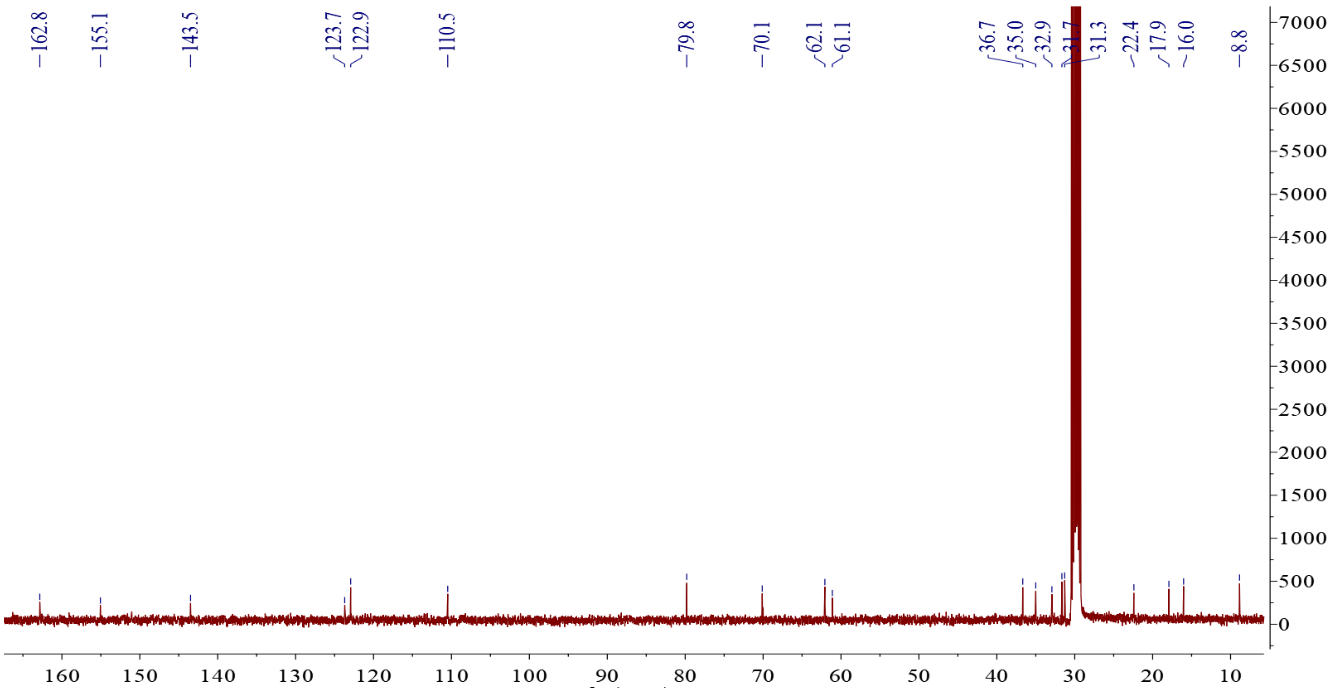

Figure S5. ^{1}H-^{1}H COSY spectrum of **1** in acetone-*d*6.

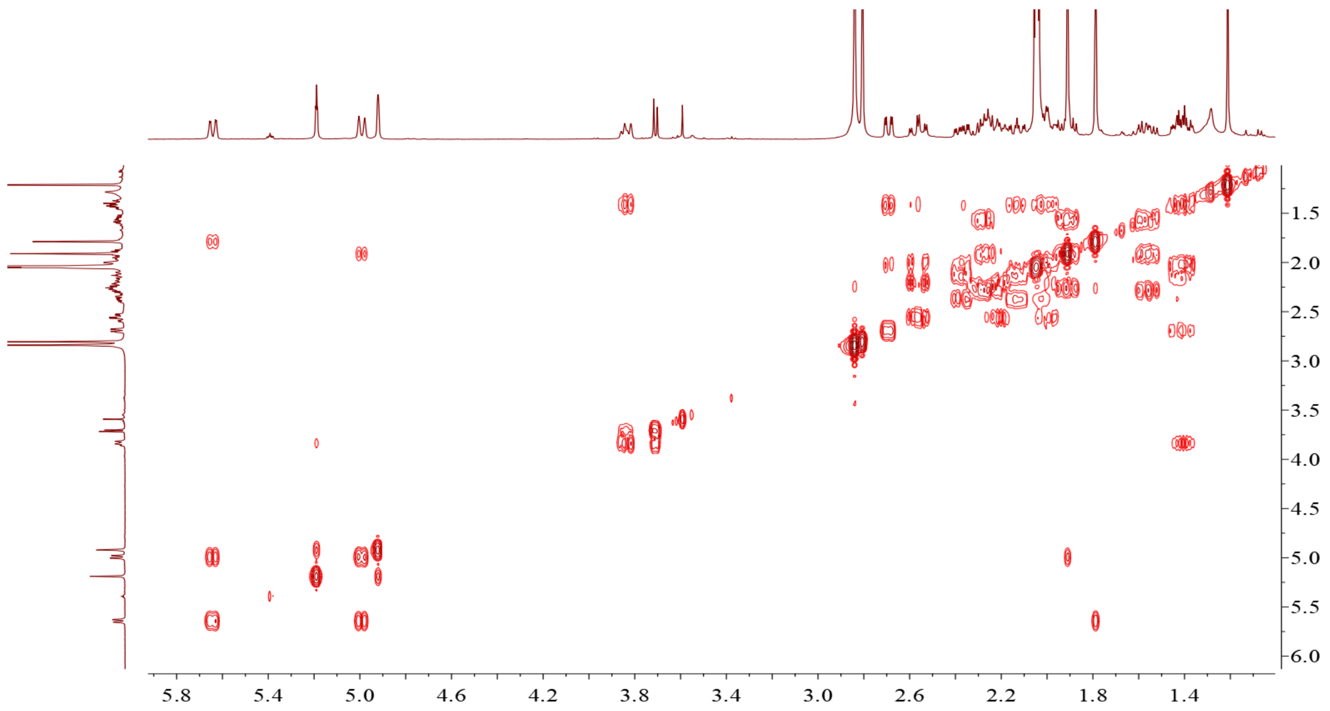

Figure S6. HSQC spectrum of **1** in acetone-*d*6.

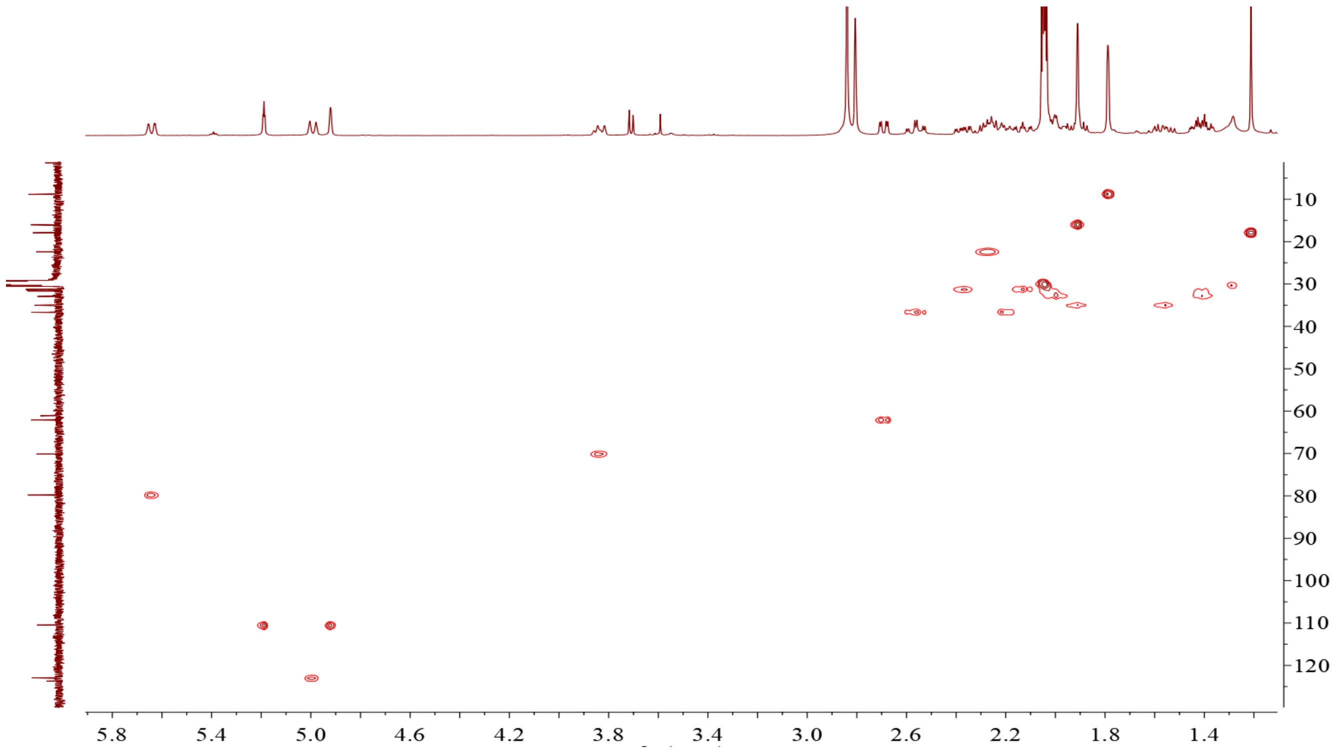

Figure S7. HMBC spectrum of **1** in acetone-d_6.

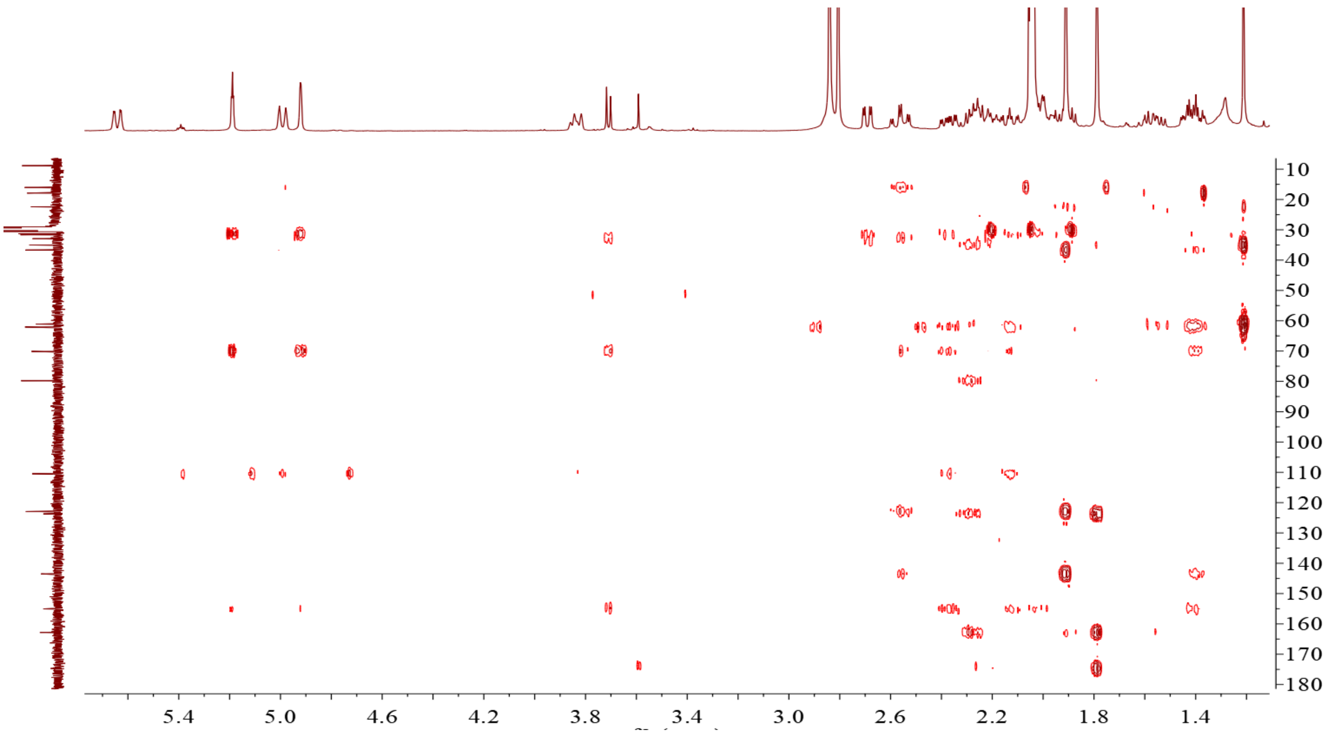

Figure S8. NOESY spectrum of **1** in acetone-d_6.

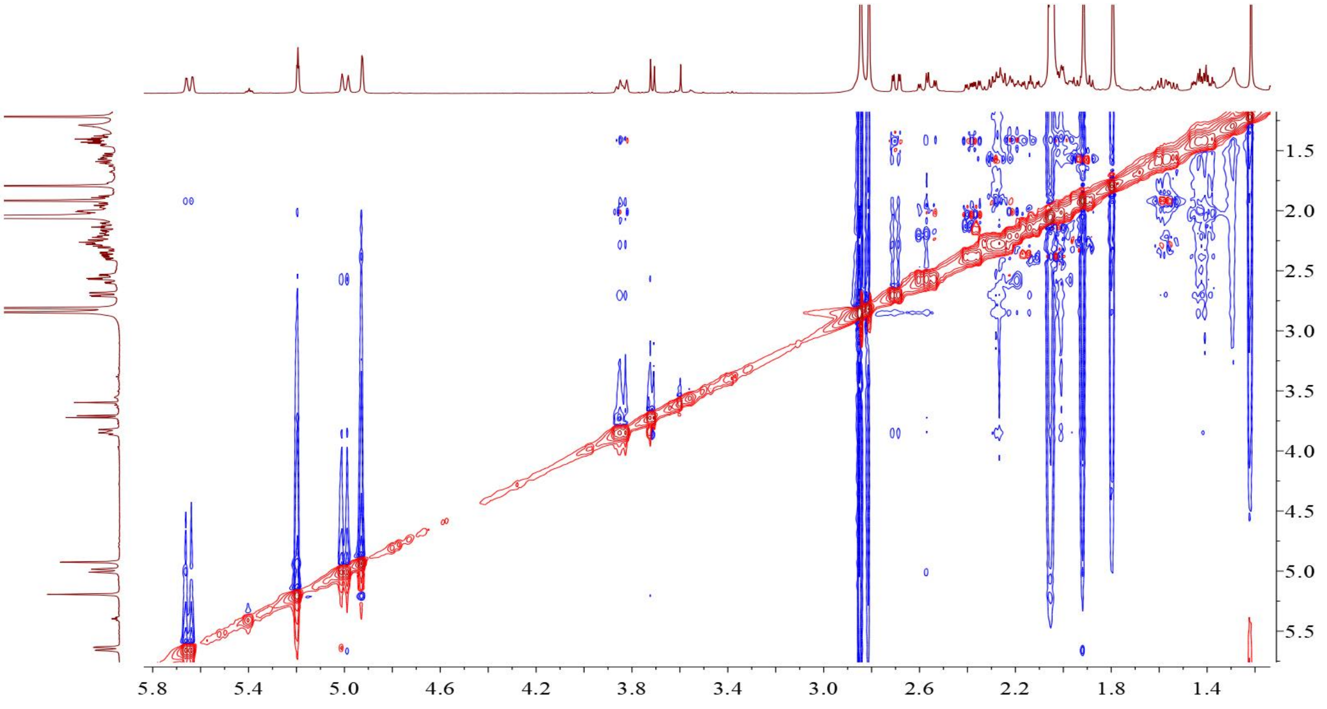

Figure S9. HR-ESIMS of **3**

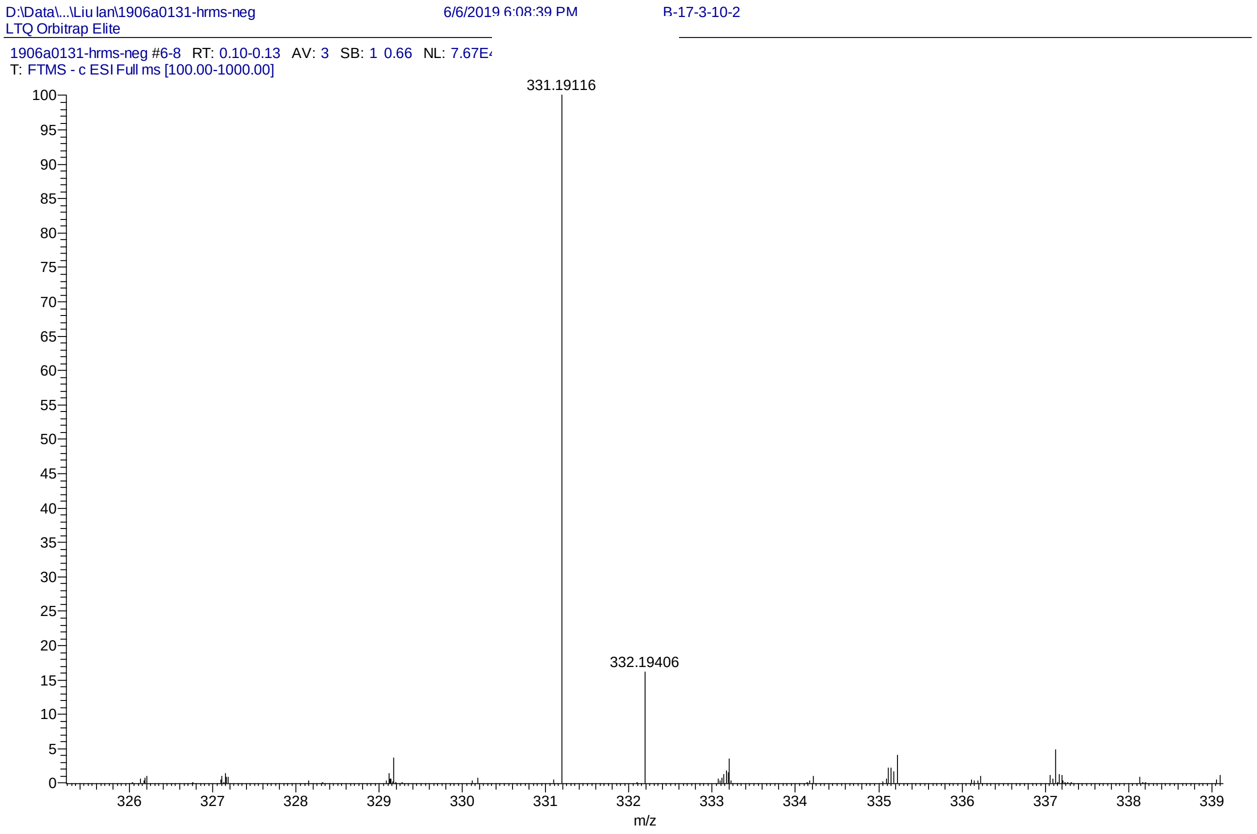

Figure S10. IR spectrum of **3**

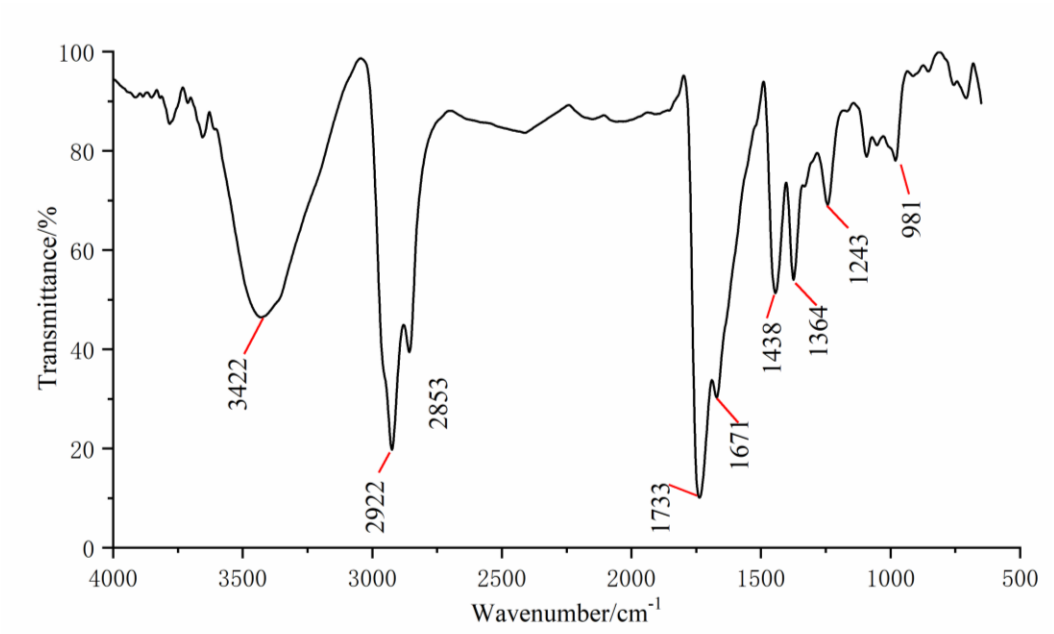

Figure S11. ^{1}H (400 MHz) NMR spectrum of **3** in $CDCl_3$

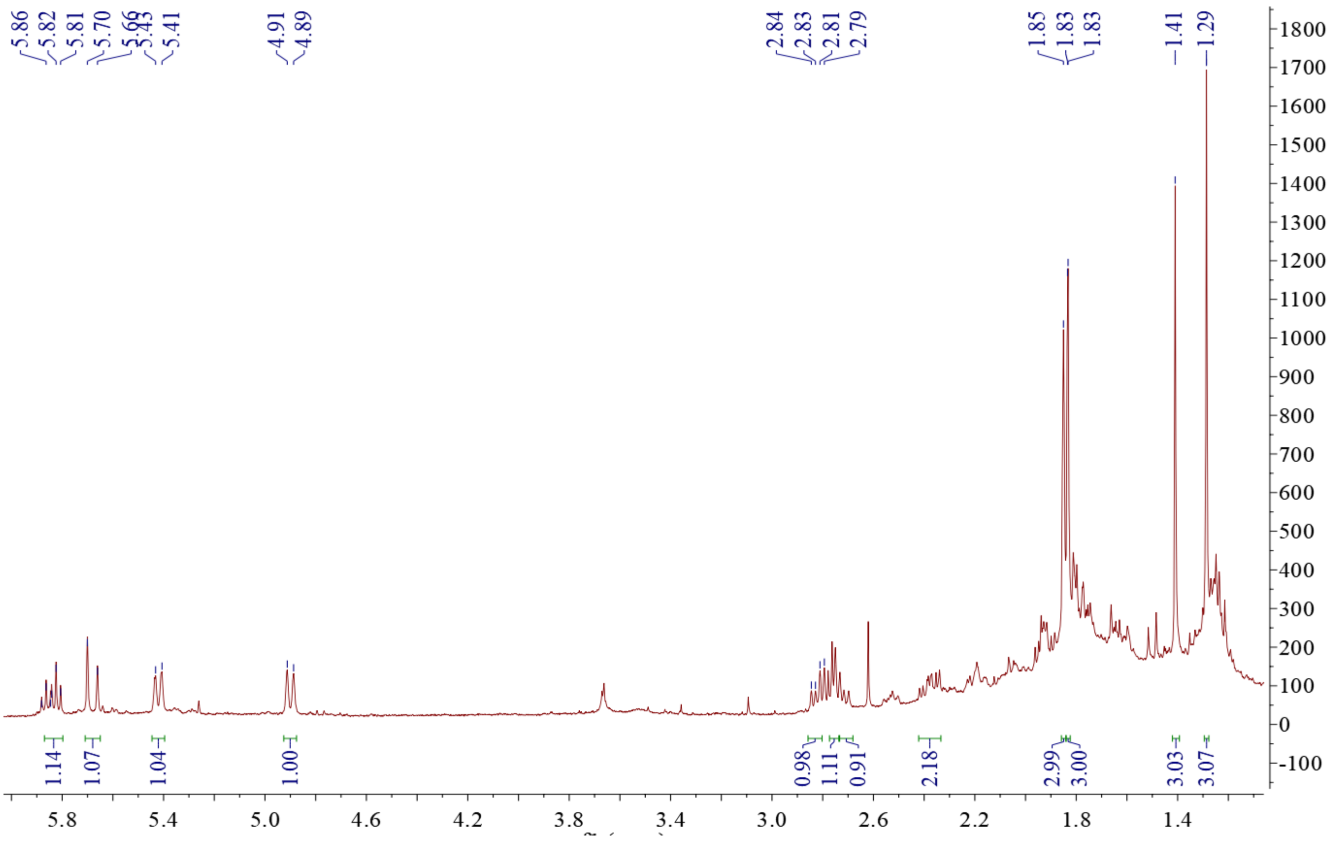

Figure S12. ^{13}C NMR spectrum of **3** in $CDCl_3$

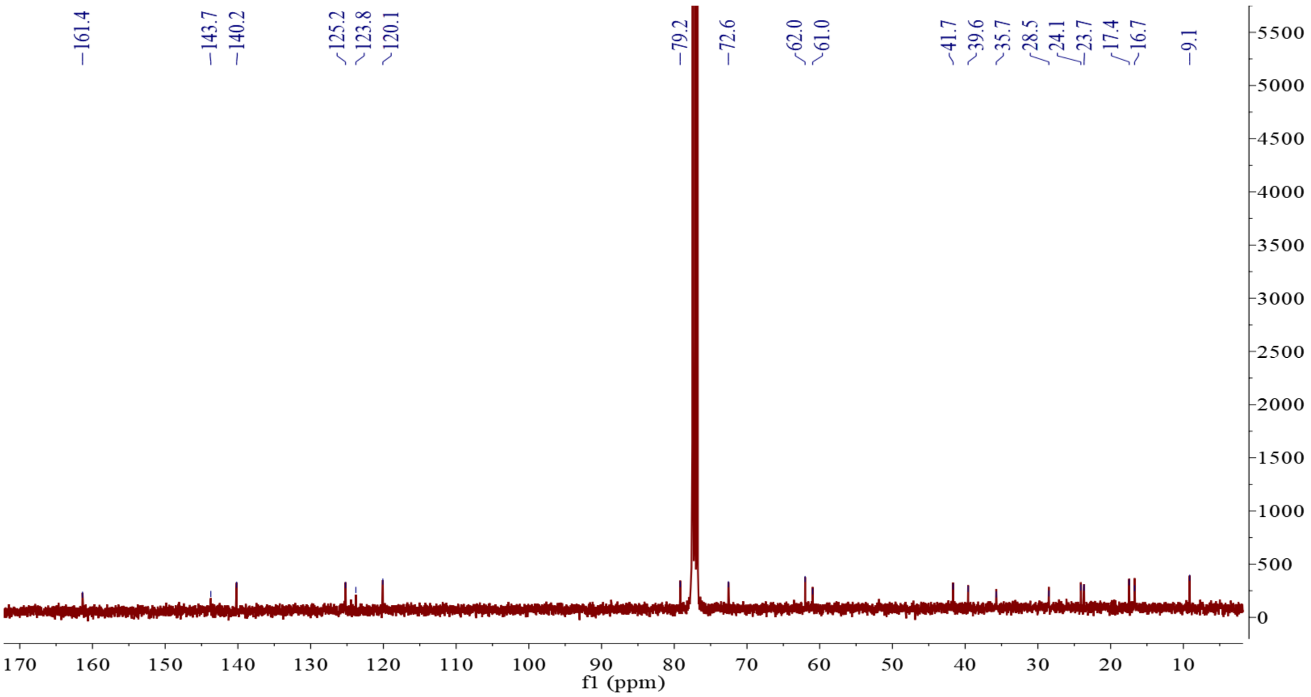

Figure S13. ^{1}H-^{1}H COSY spectrum of **3** in $CDCl_3$

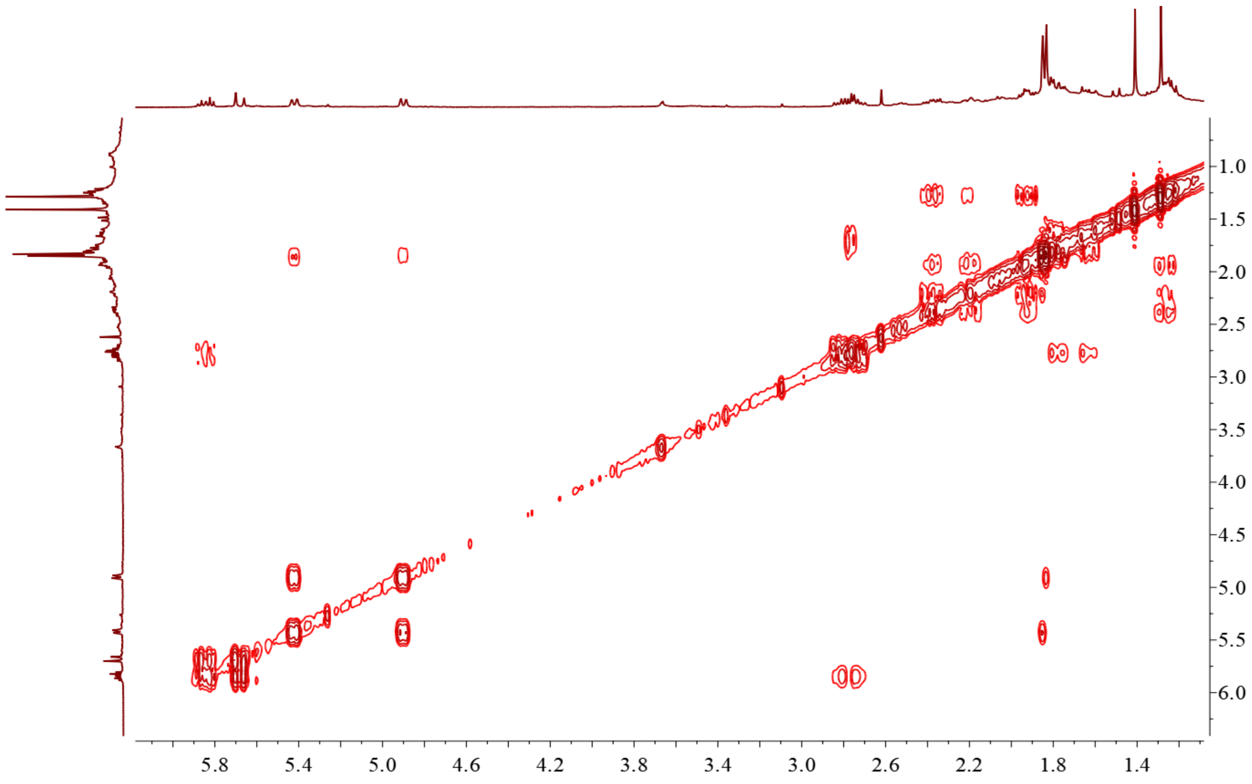

Figure S14. HSQC spectrum of **3** in $CDCl_3$

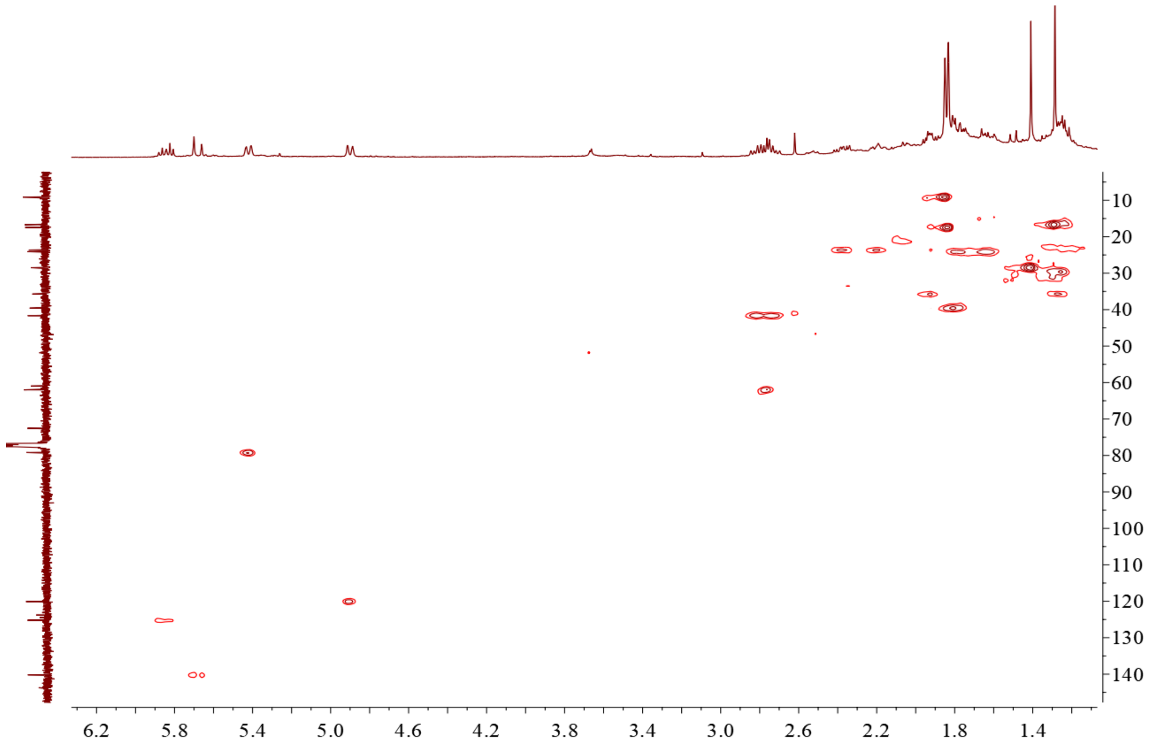

Figure S15. HMBC spectrum of **3** in $CDCl_3$

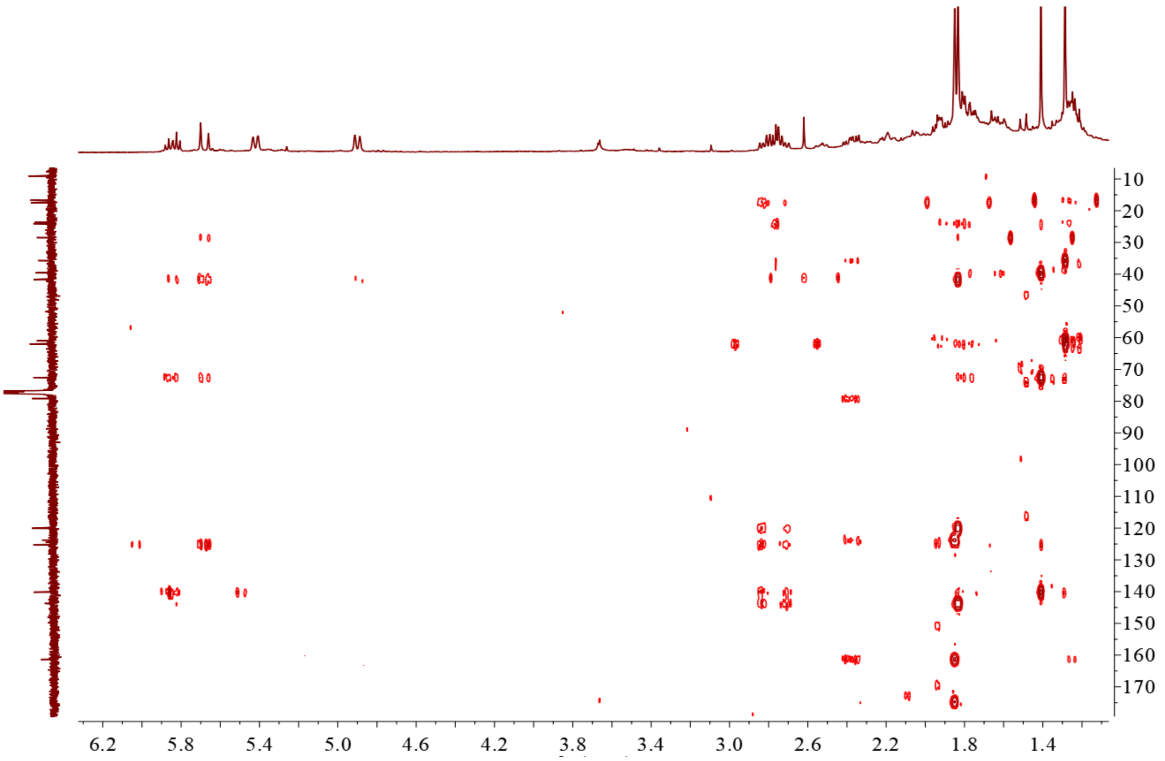

Figure S16. NOESY spectrum of **3** in $CDCl_3$.

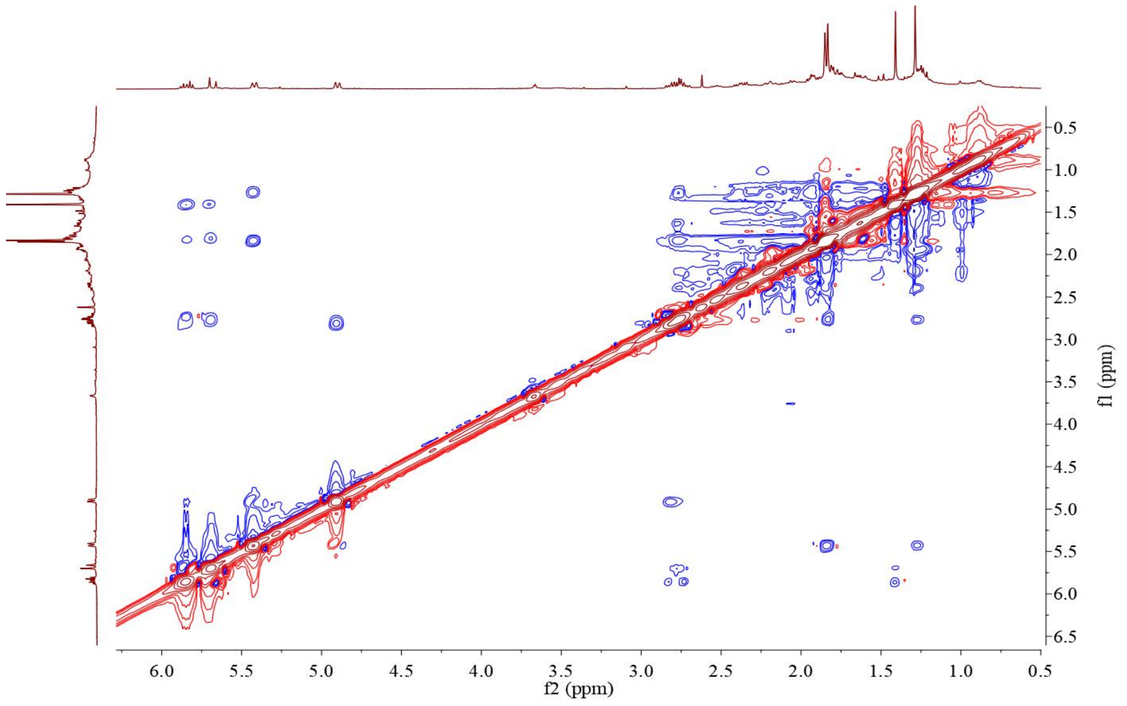

Figure S17. HR-ESIMS of **10**

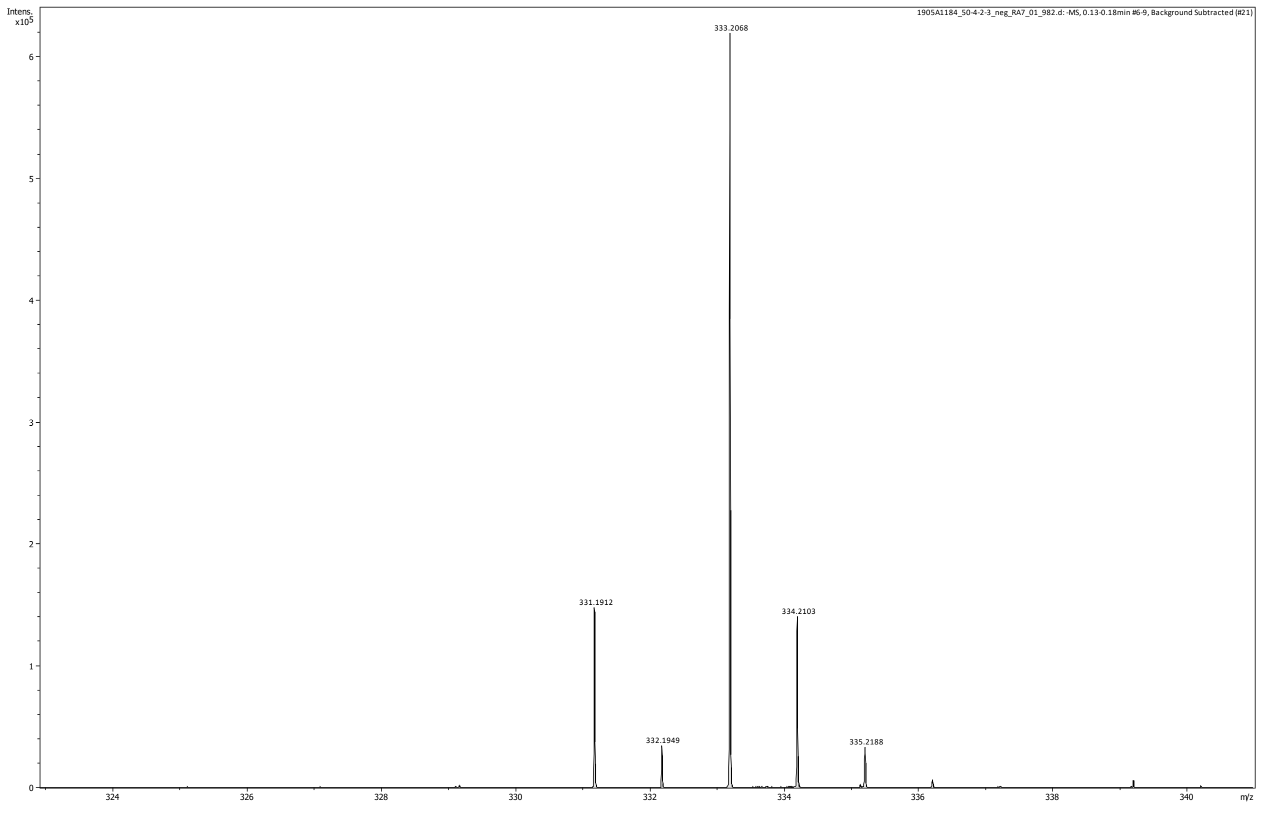

Figure S18. IR spectrum of **10**

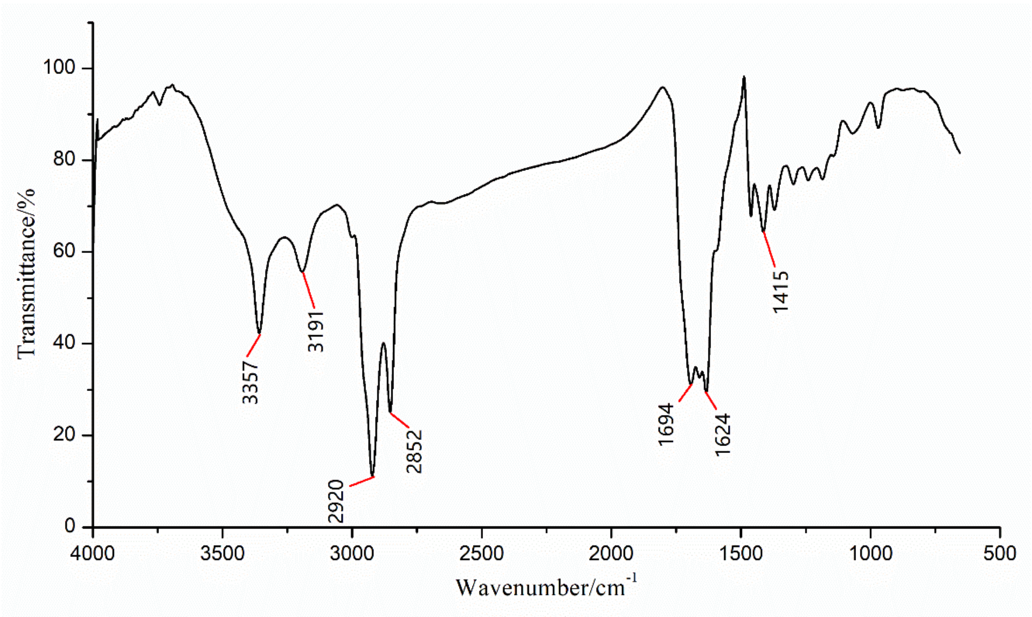

Figure S19. ¹H (400 MHz) NMR spectrum of **10** in MeOH-d_4.

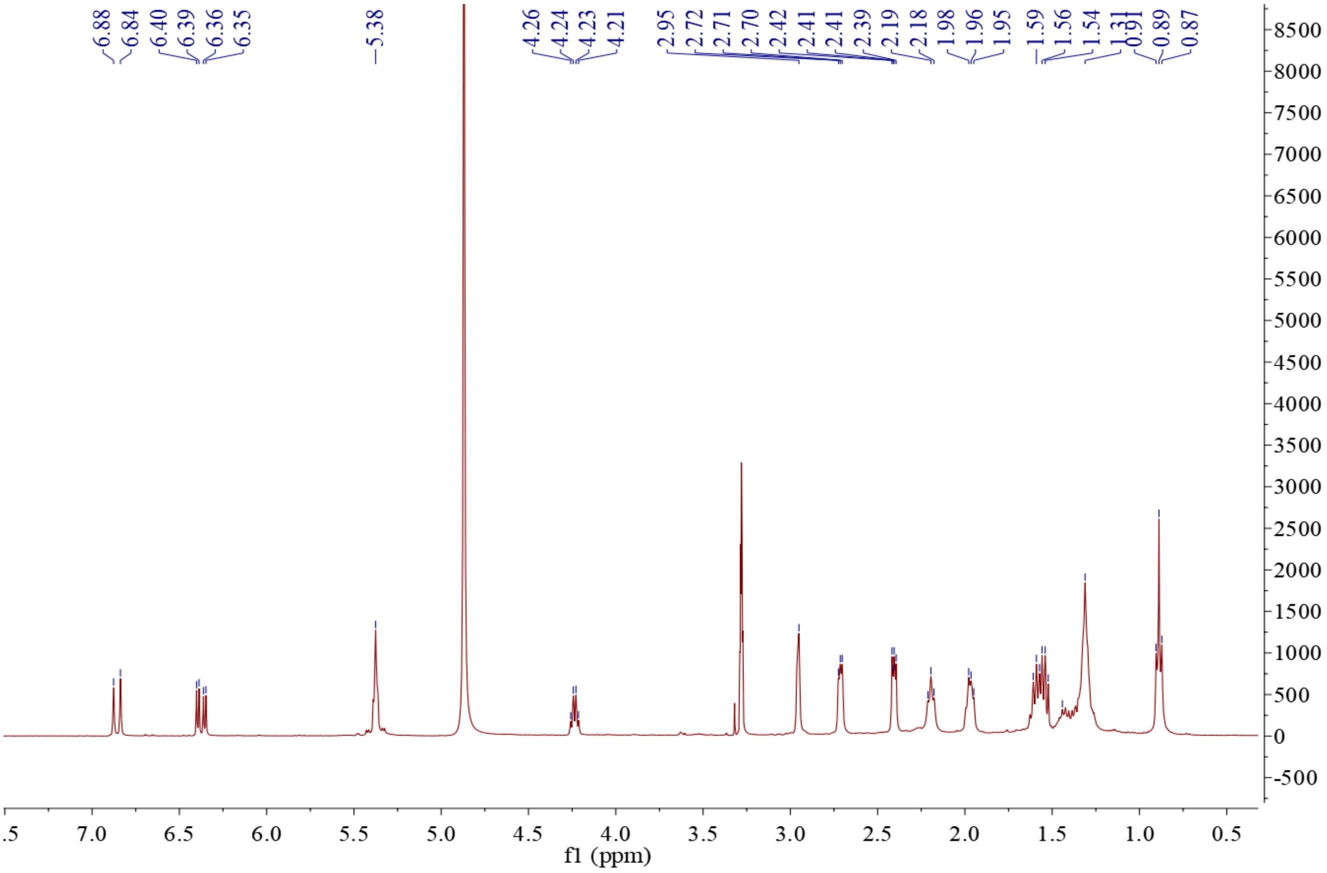

Figure S20. ^{13}C NMR spectrum of **10** in MeOH-d_4.

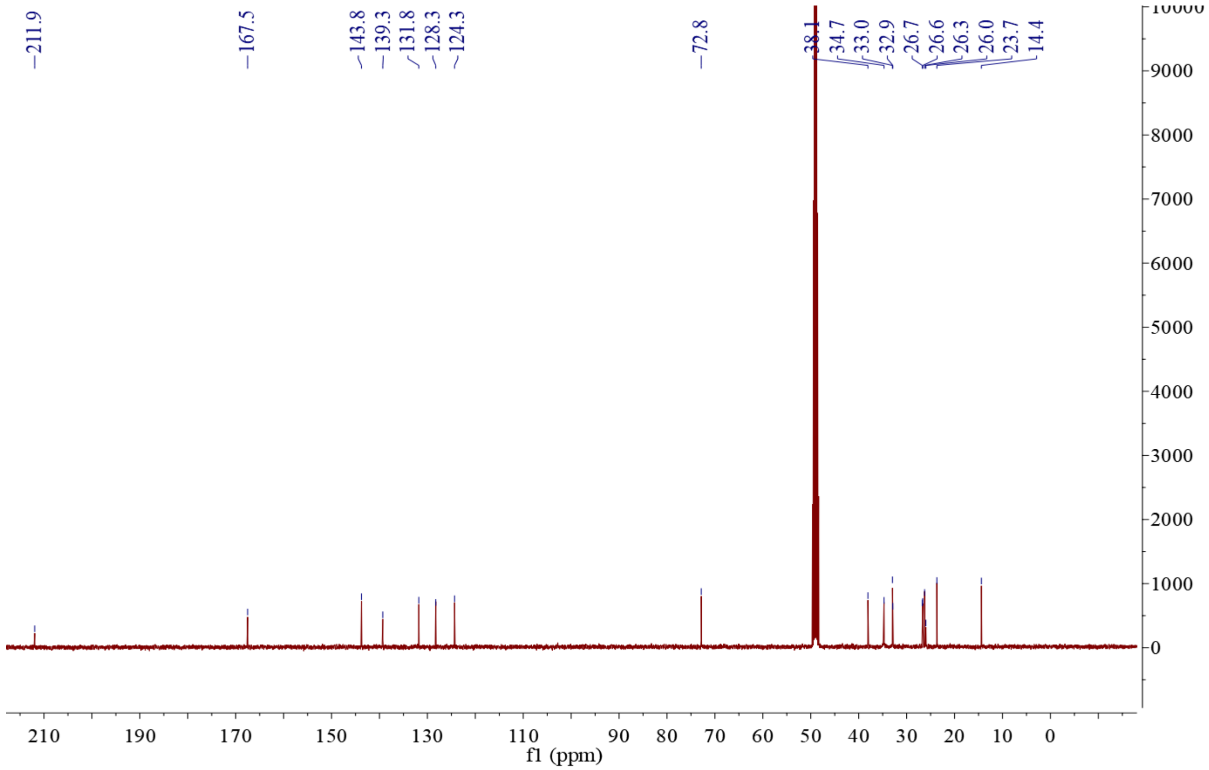

Figure S21. ^{1}H-^{1}H COSY spectrum of **10** in MeOH-d_4.

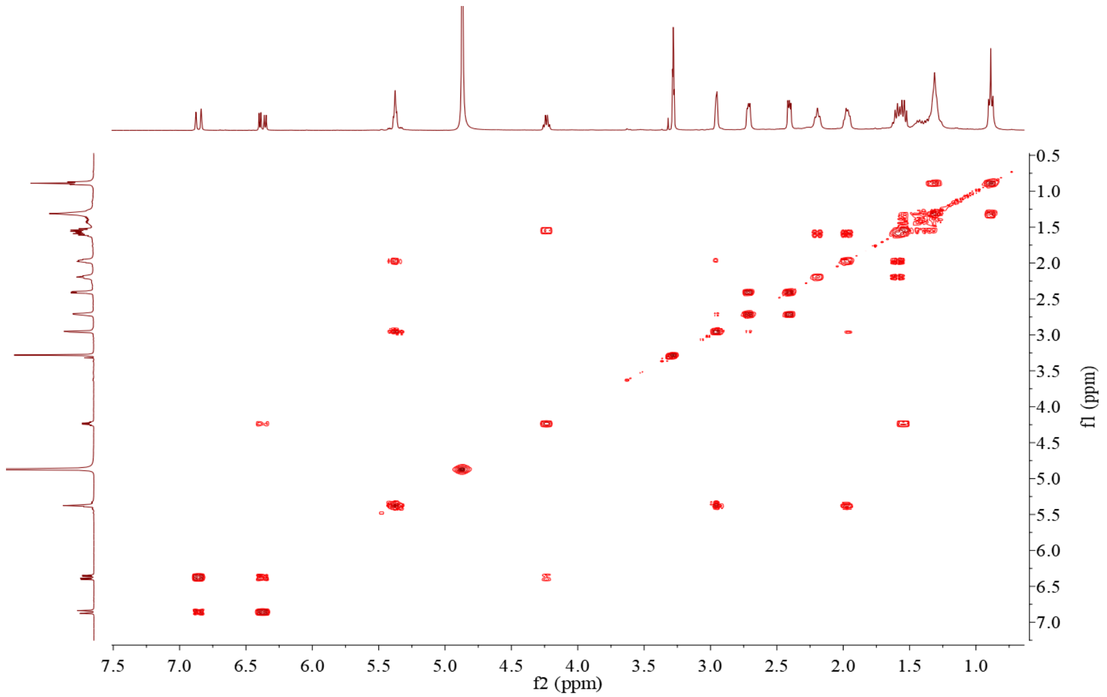

Figure S22. HSQC spectrum of **10** in MeOH-d_4.

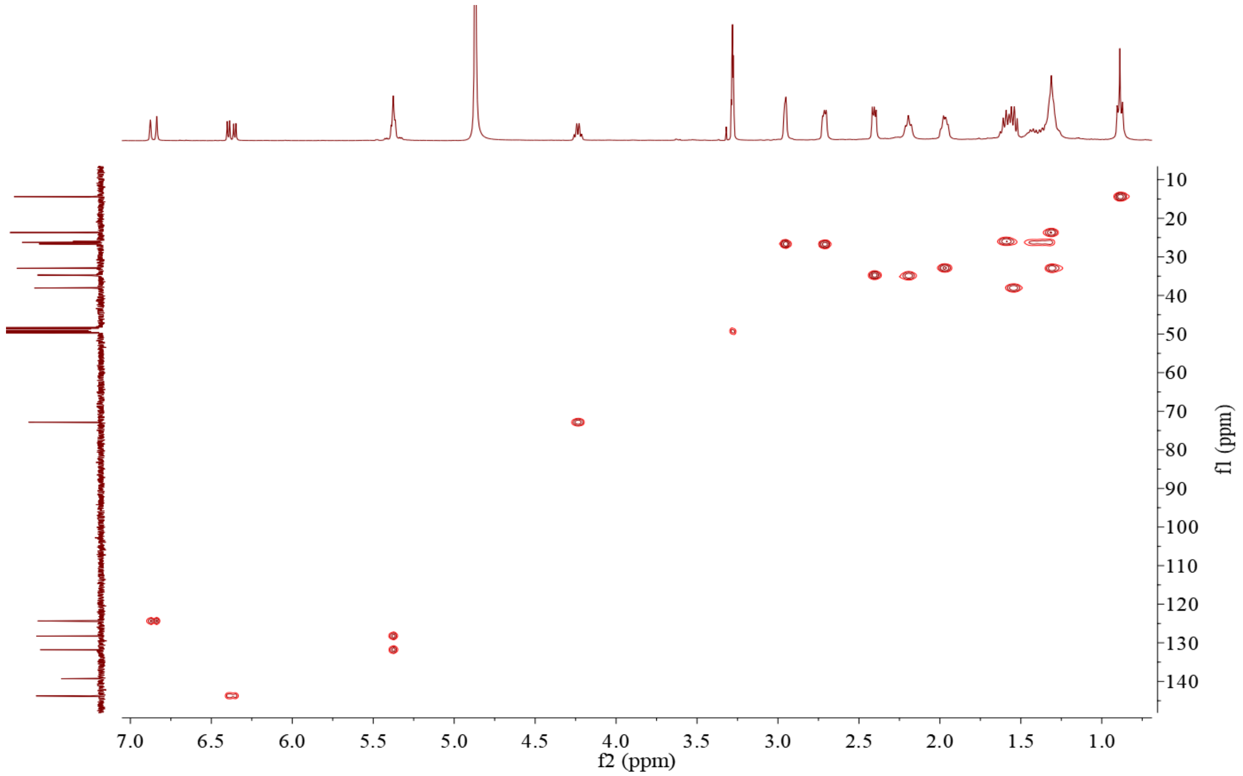

Figure S23. HMBC spectrum of **10** in MeOH-d_4

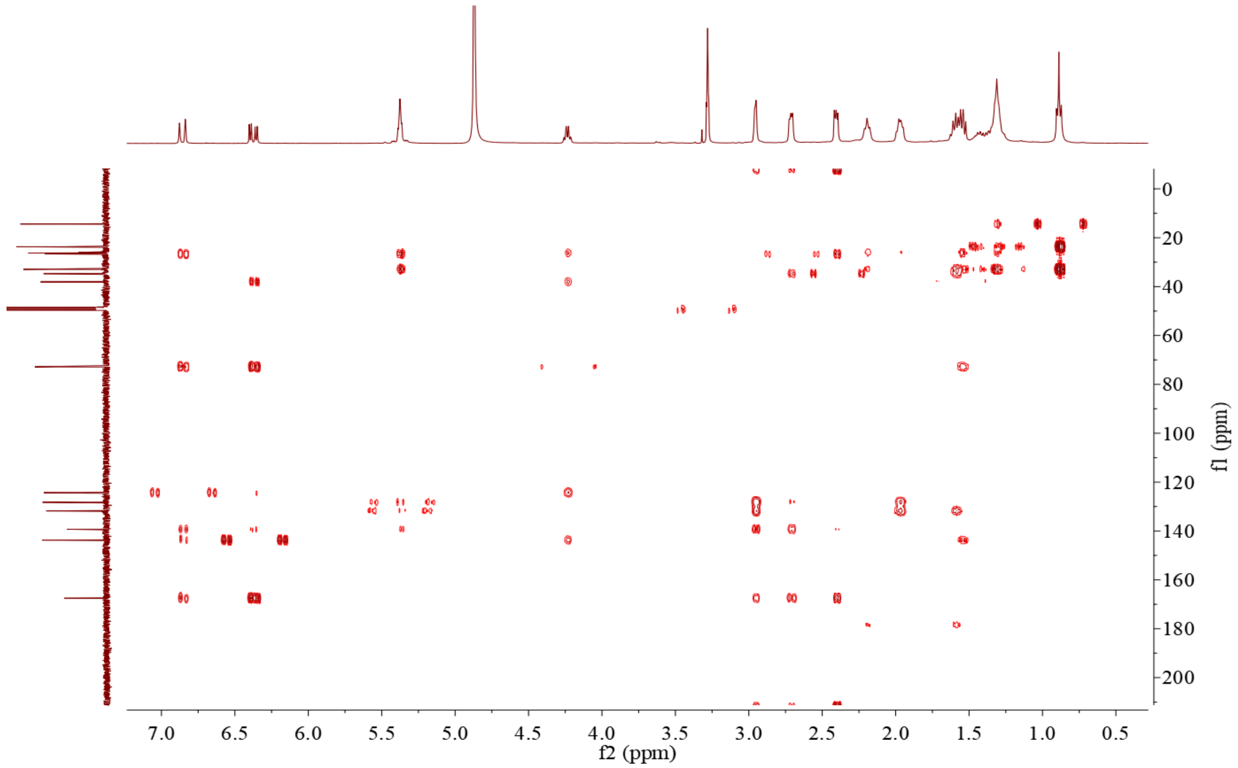

Figure S24. NOESY spectrum of **10** in MeOH-d_4

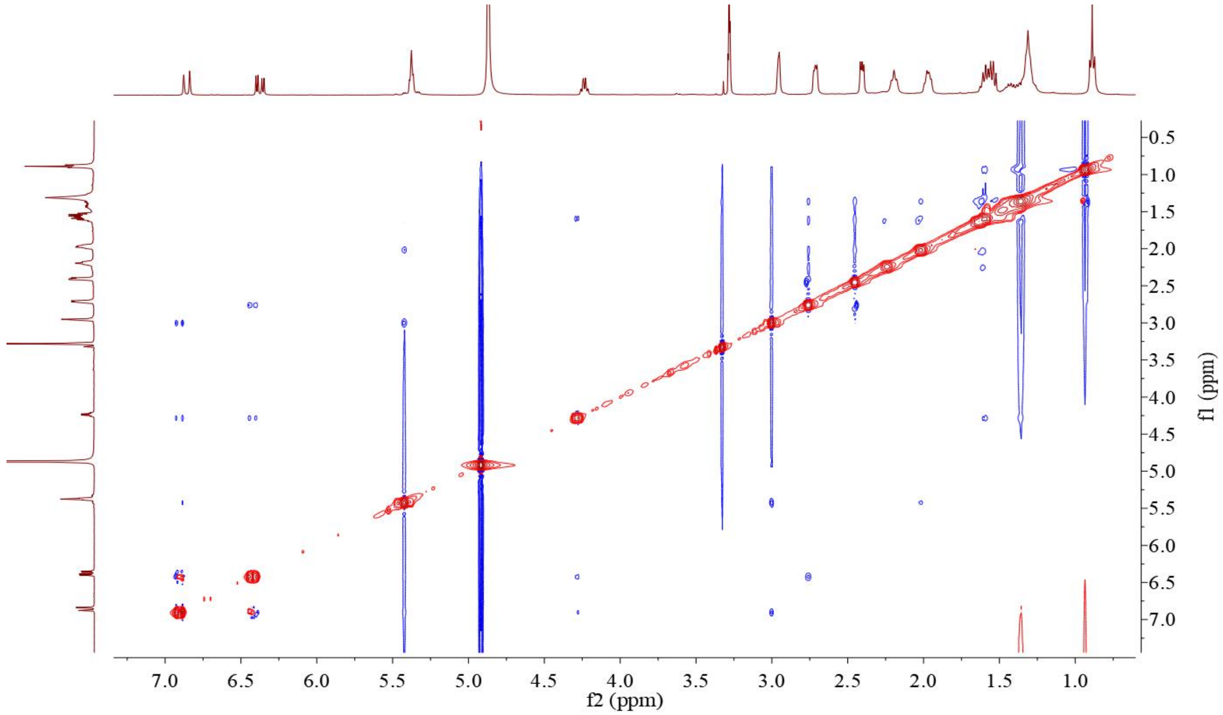

Figure S25. HR-ESIMS of **11**

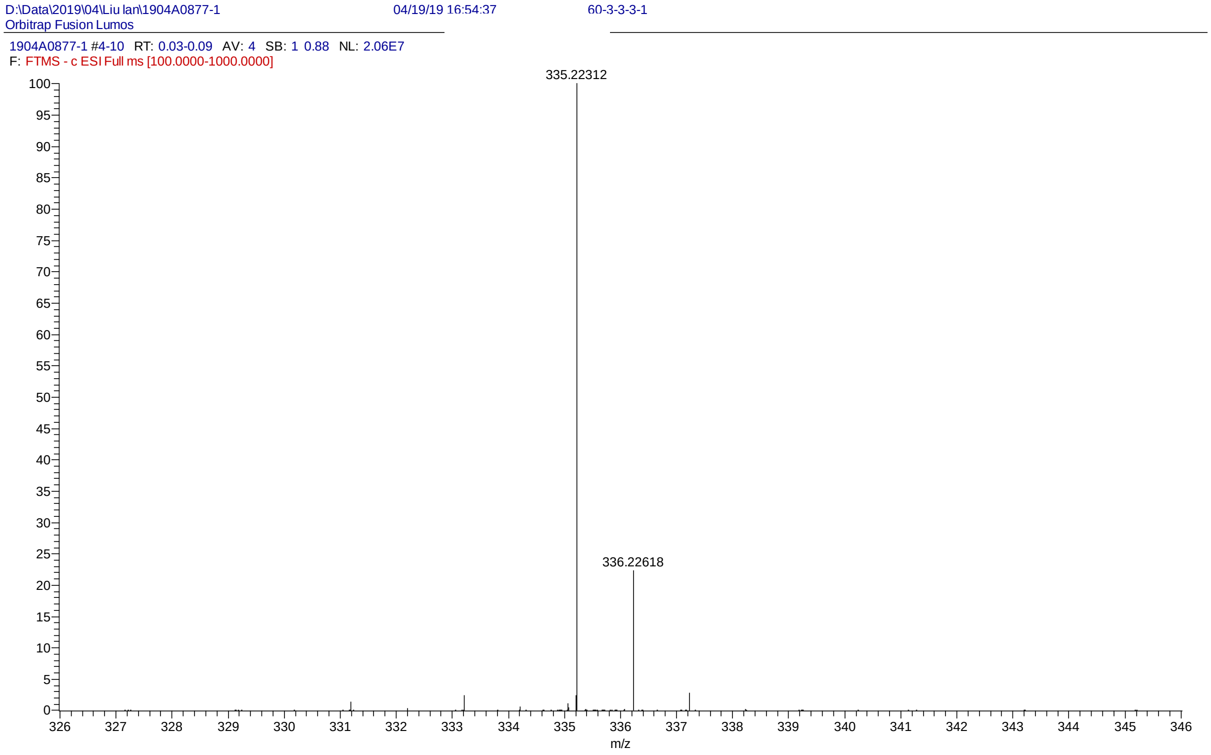

Figure S26. IR spectrum of **11**

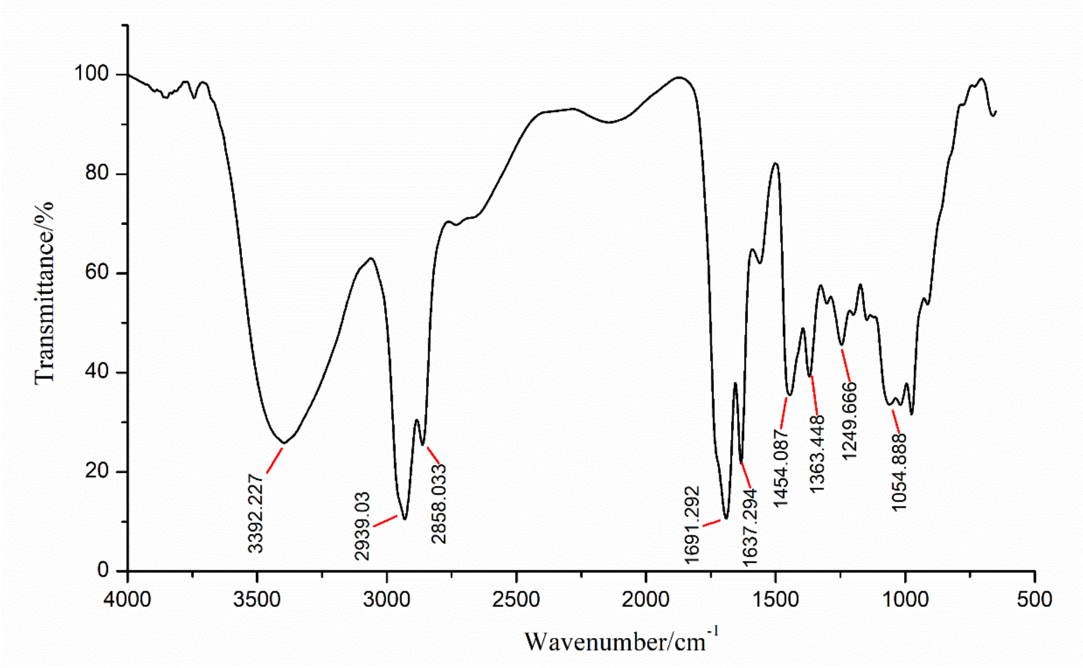

Figure S27. ^{1}H (400 MHz) NMR spectrum of **11** in MeOH-*d*4

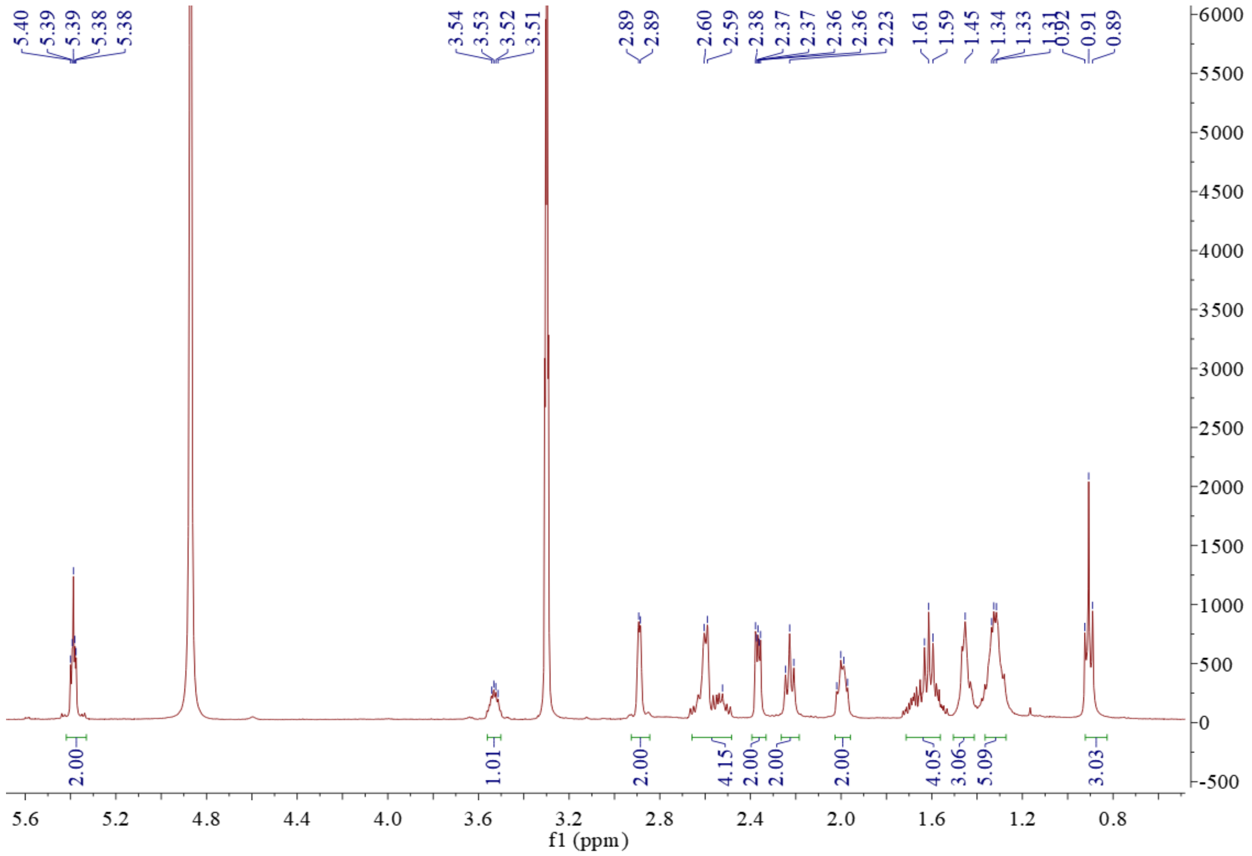

Figure S28. ^{13}C NMR spectrum of **11** in MeOH-*d*4

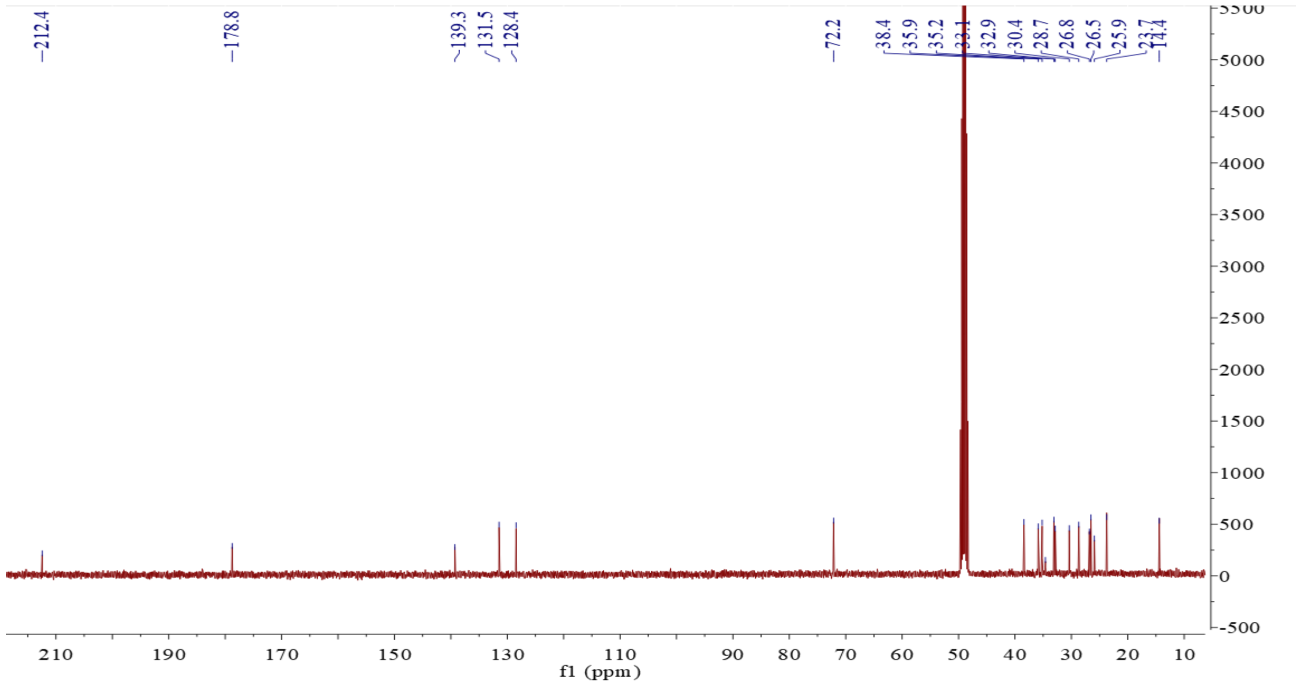

Figure S29. ^{1}H-^{1}H COSY spectrum of **11** in MeOH-d_4.

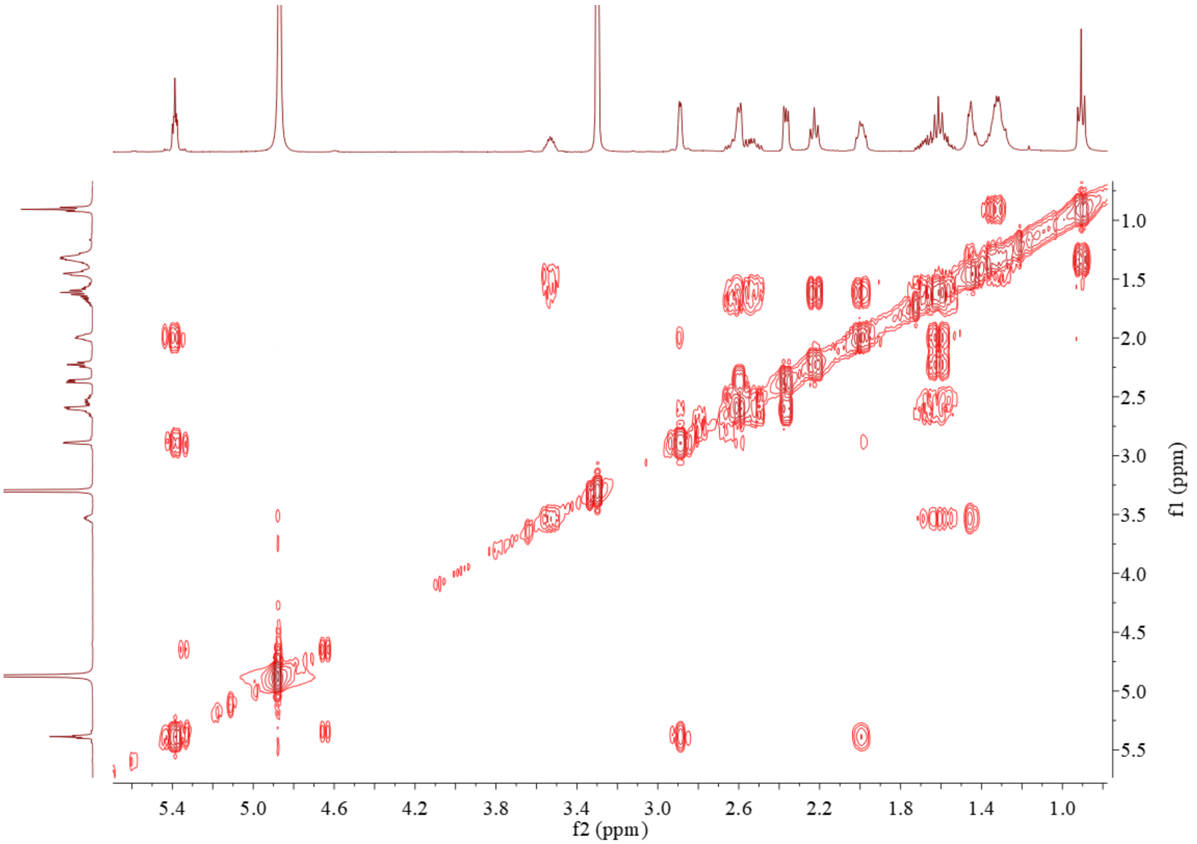

Figure S30. HSQC spectrum of **11** in MeOH-d_4.

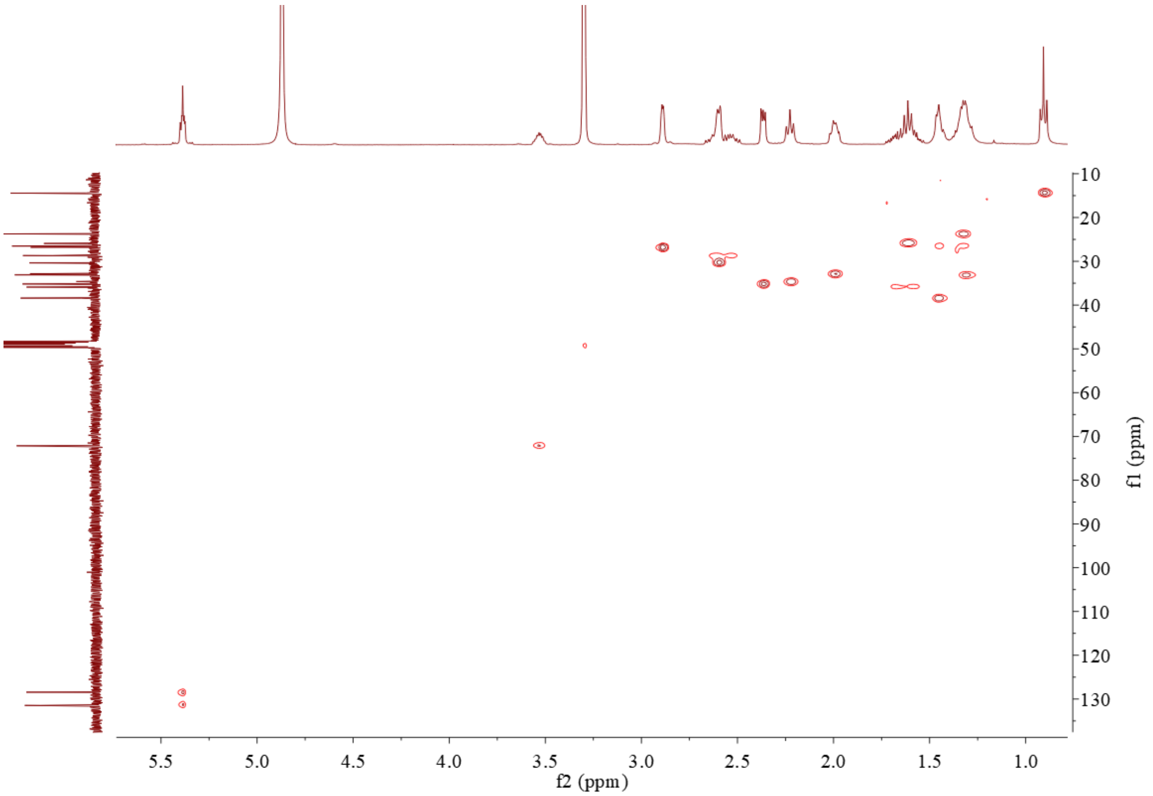

Figure S31. HMBC spectrum of **11** in MeOH-*d*4

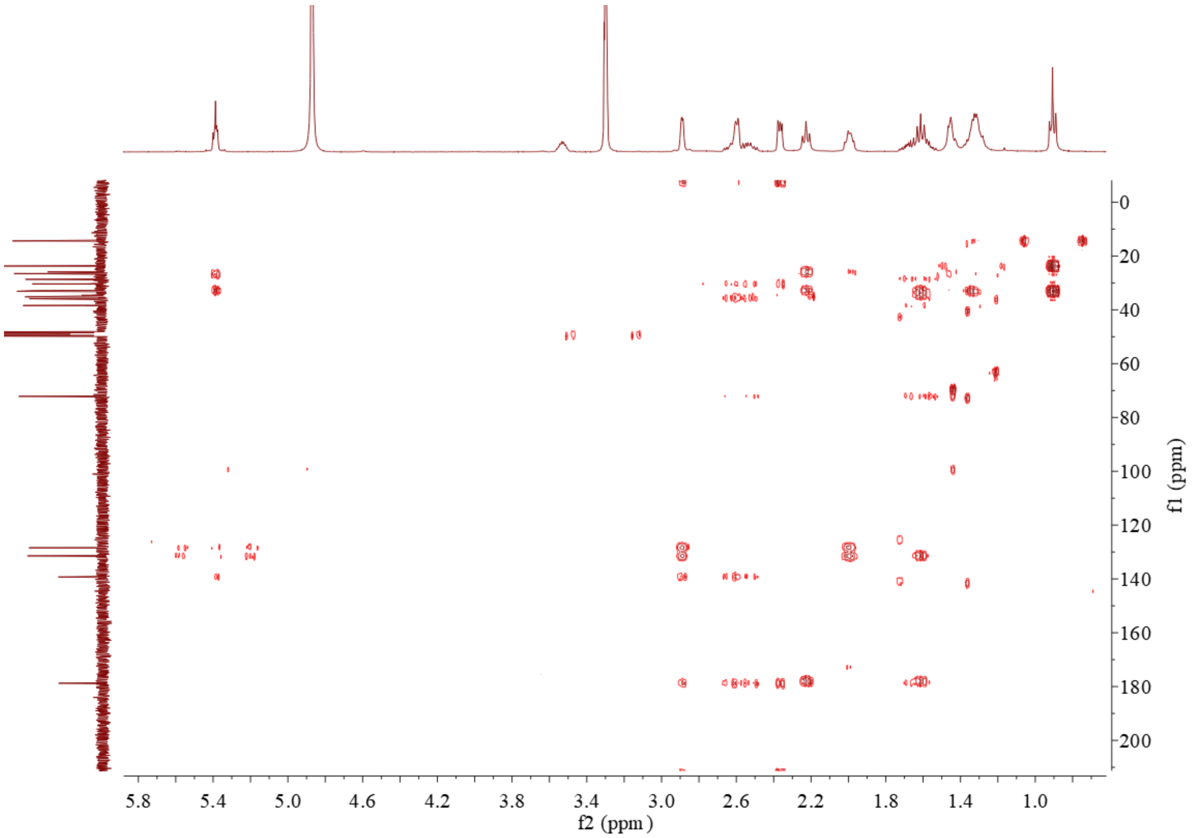

Figure S32. HR-ESIMS of **12**

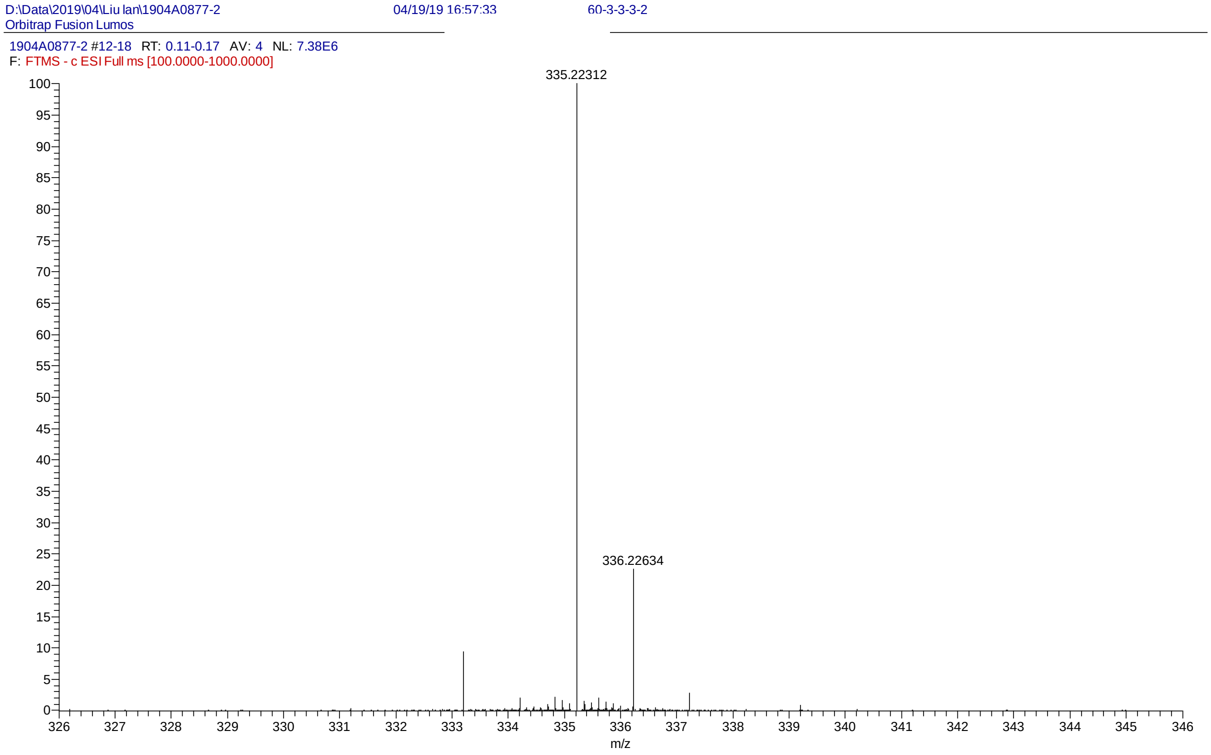

Figure S33. IR spectrum of **12**

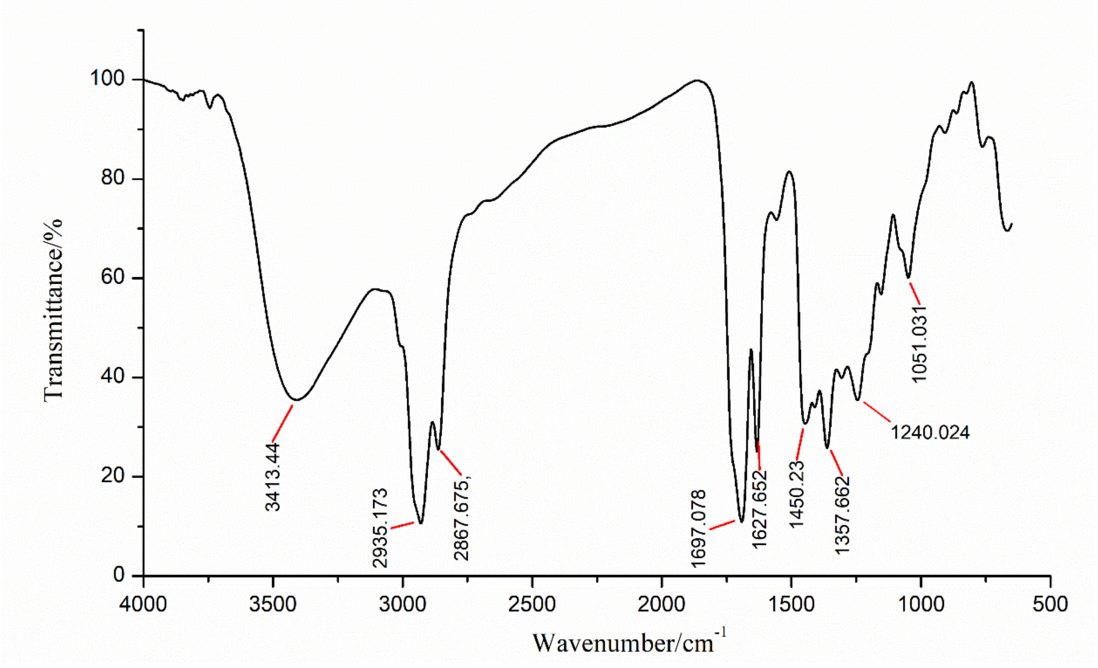

Figure S34. ^{1}H (400 MHz) NMR spectrum of **12** in MeOH-*d*$_4$

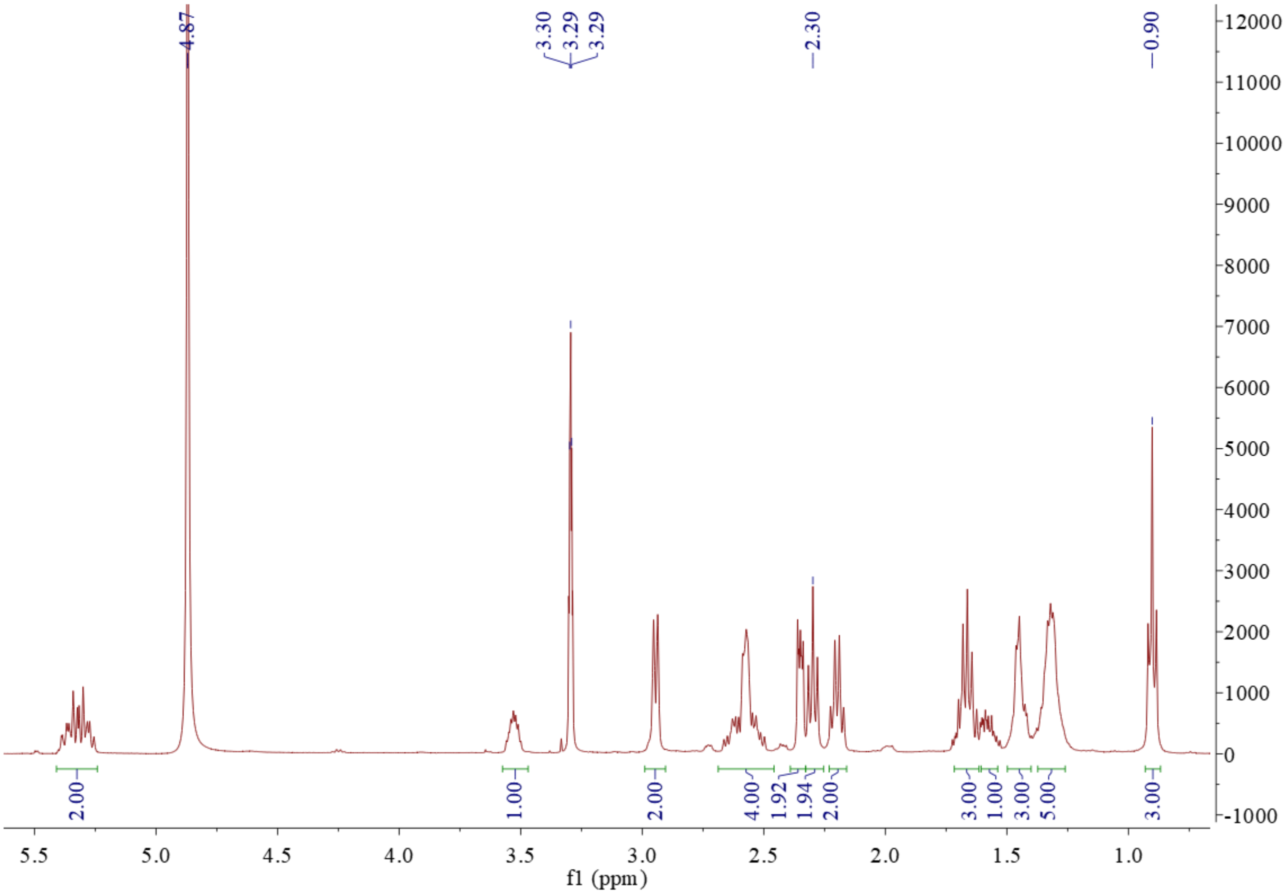

Figure S35. ^{13}C NMR spectrum of **11** in MeOH-*d*$_4$

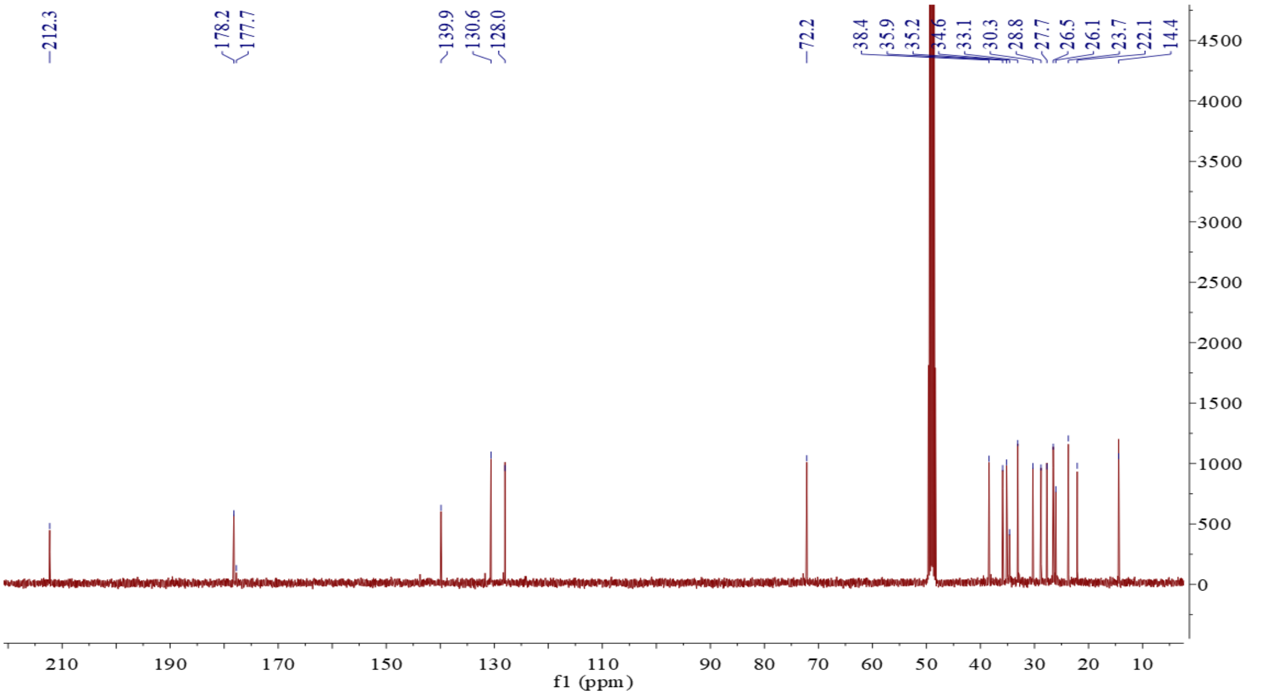

Figure S36. ^{1}H-^{1}H COSY spectrum of **12** in MeOH-*d*4

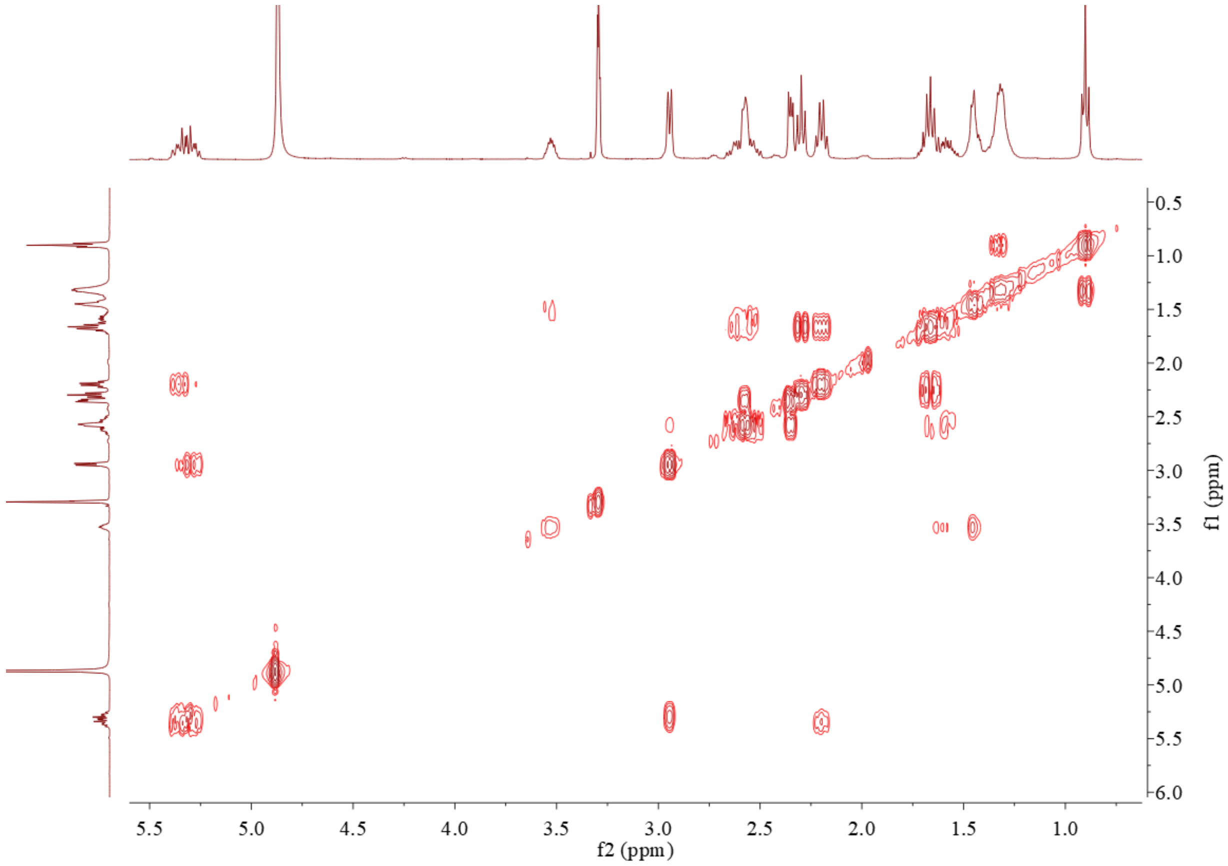

Figure S37. HSQC spectrum of **12** in MeOH-*d*4

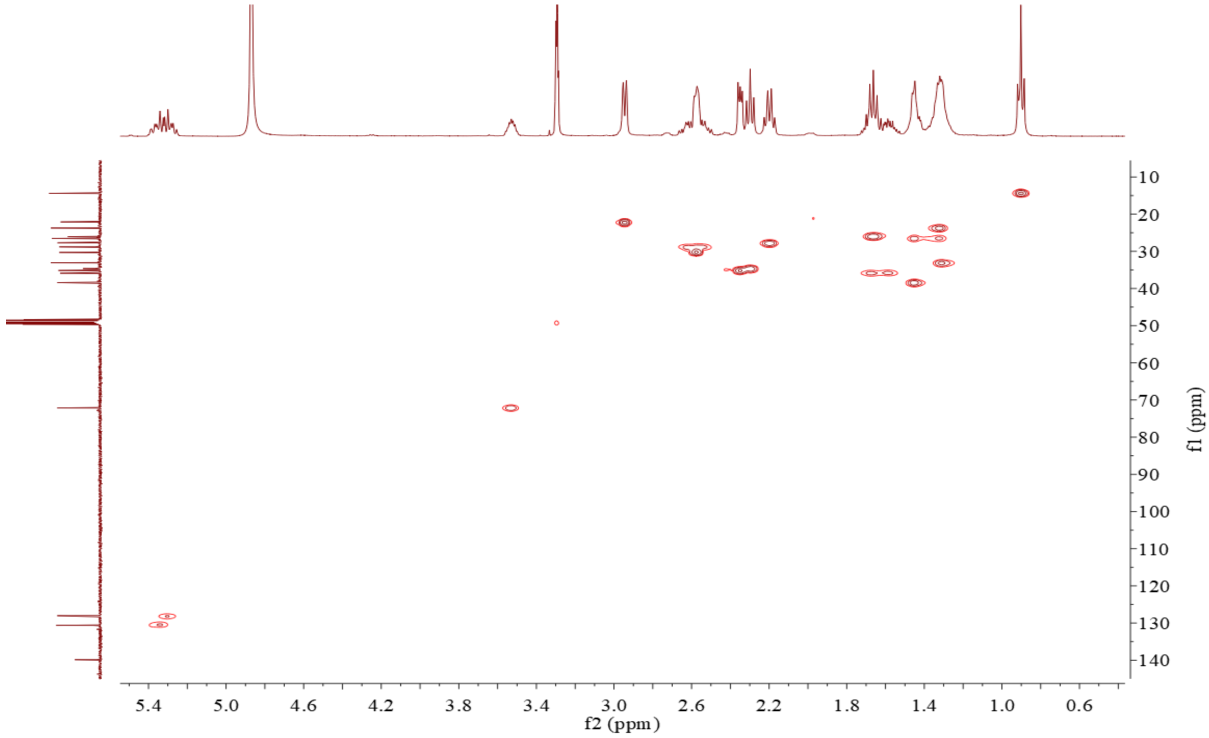

Figure S38. HMBC spectrum of **12** in MeOH-d_4

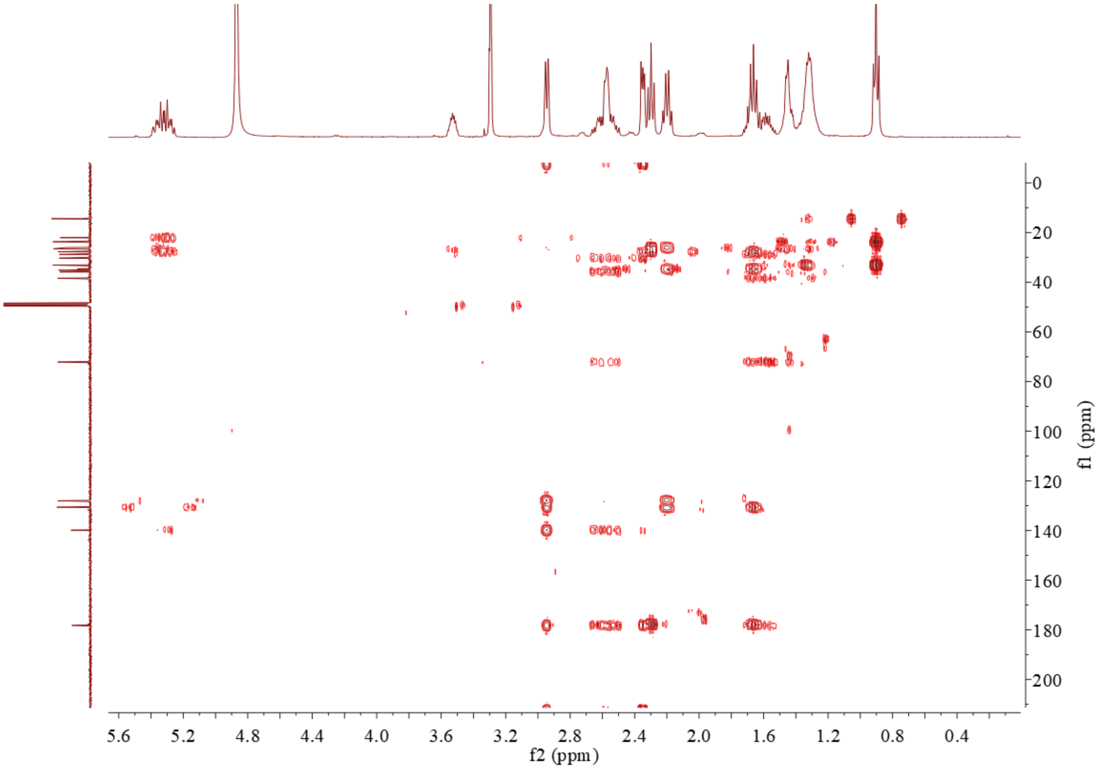

Figure S39. NOESY spectrum of **12** in MeOH-d_4

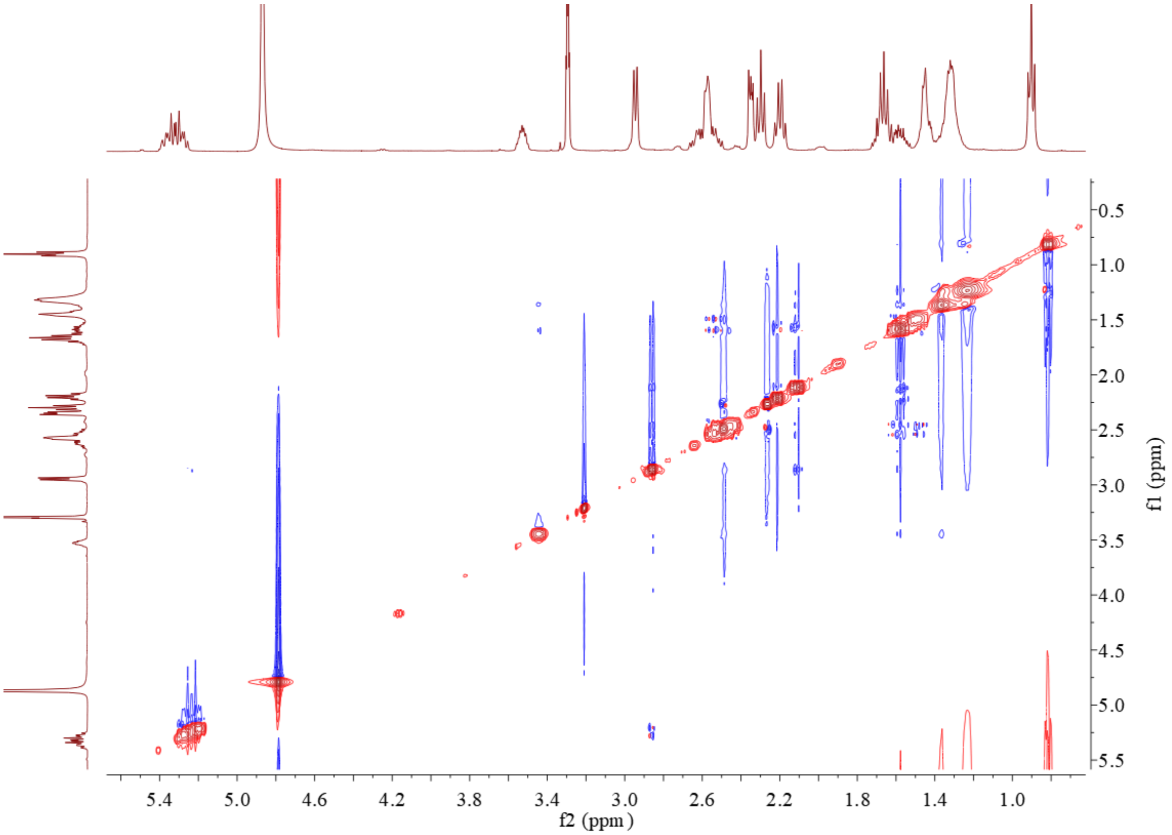

Figure S40. HR-ESIMS of **13**

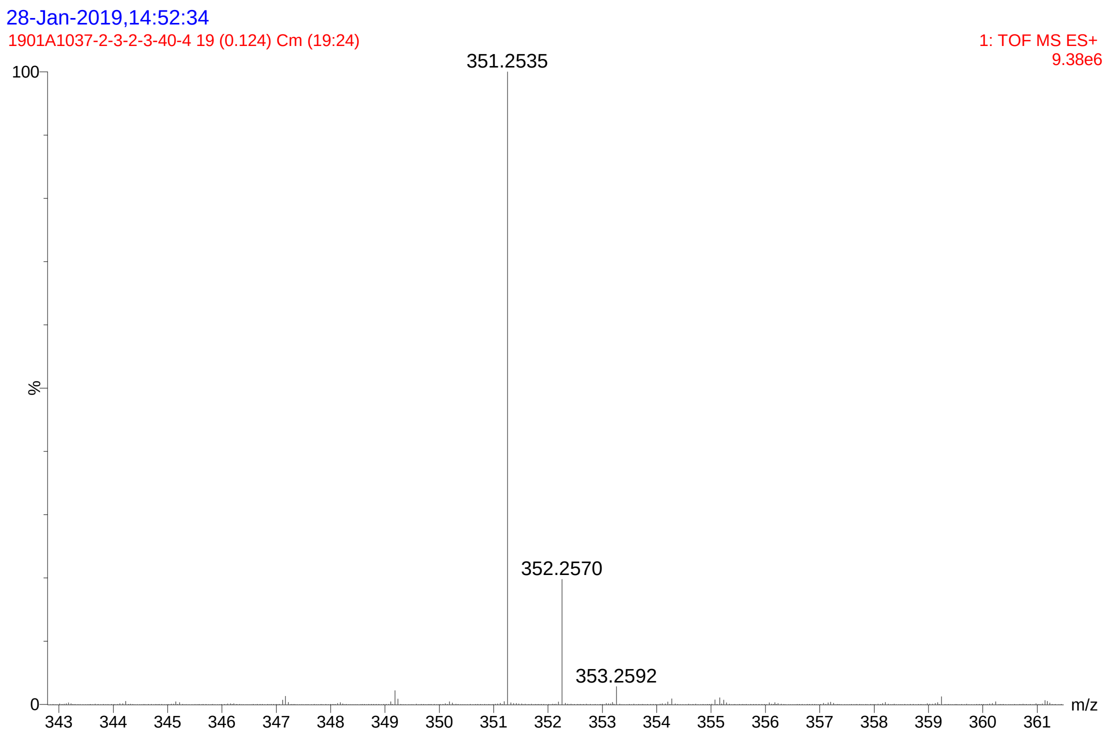

Figure S41. IR spectrum of **13**

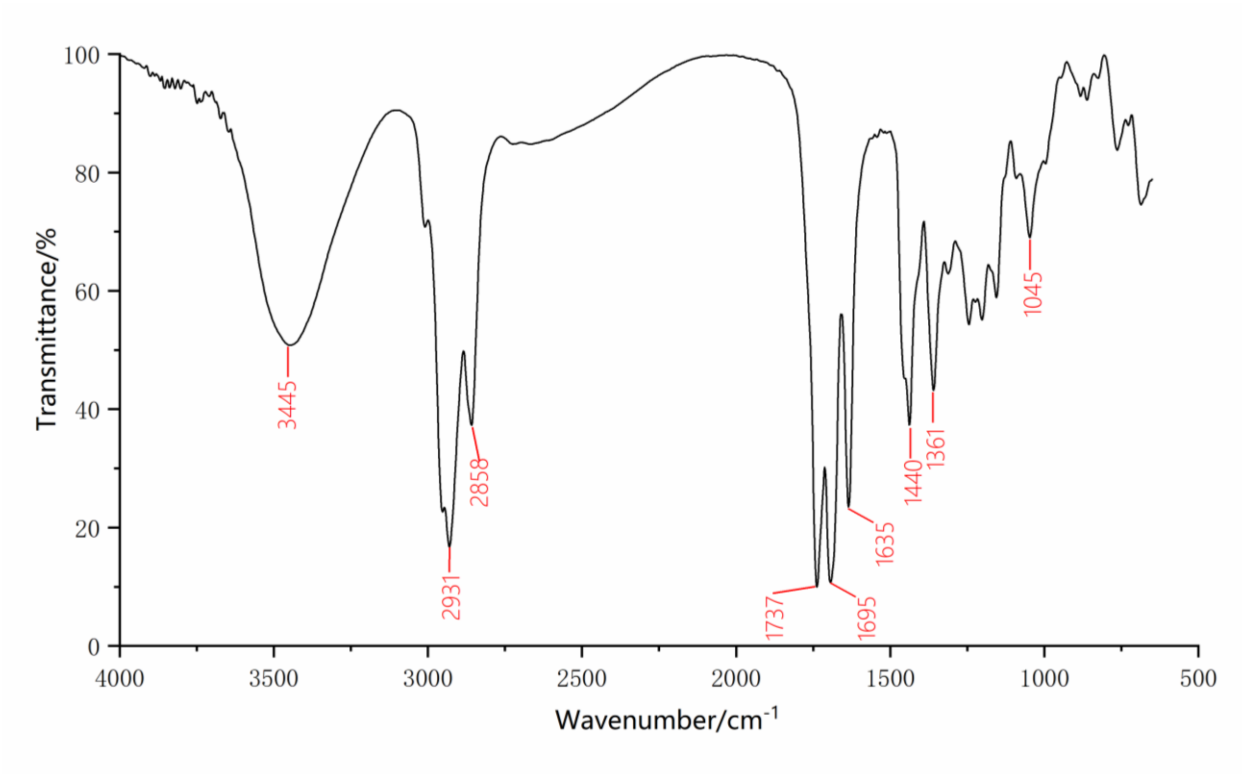

Figure S42. ^{1}H (400 MHz) NMR spectrum of **13** in acetone-*d*6.

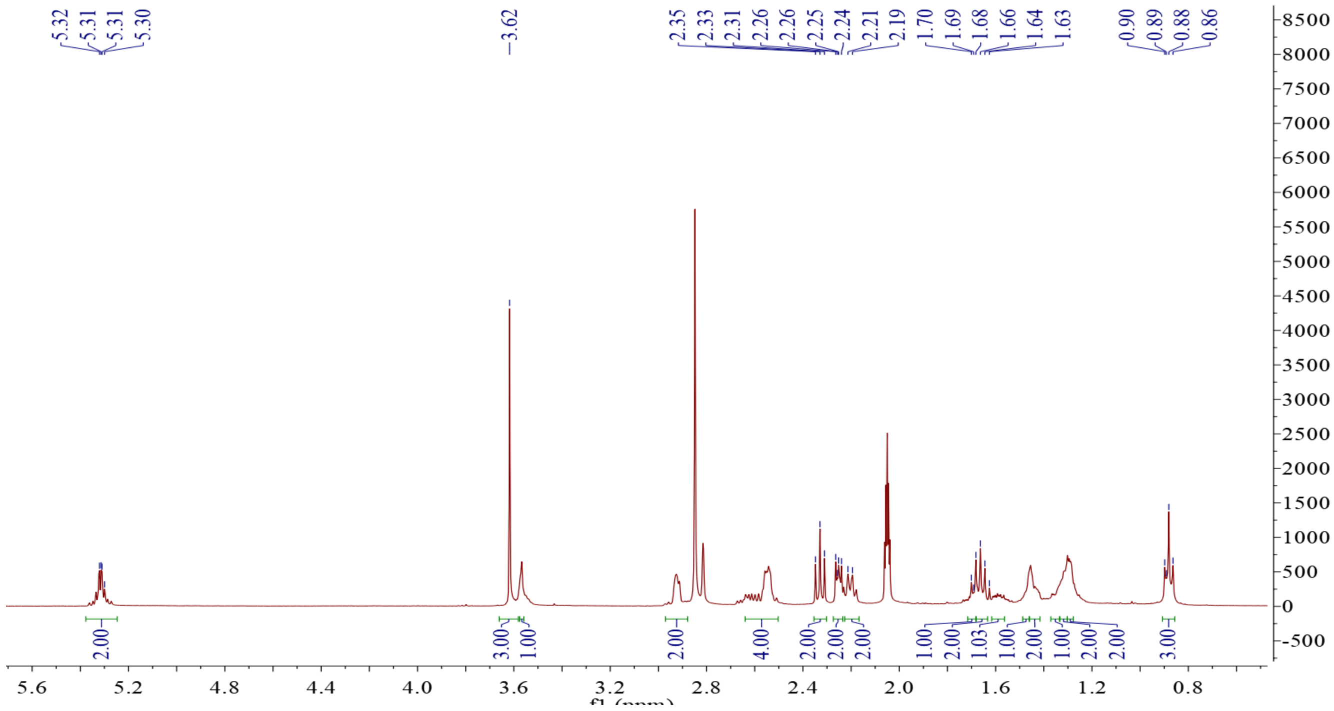

Figure S43. ^{13}C NMR spectrum of **13** in acetone-*d*6.

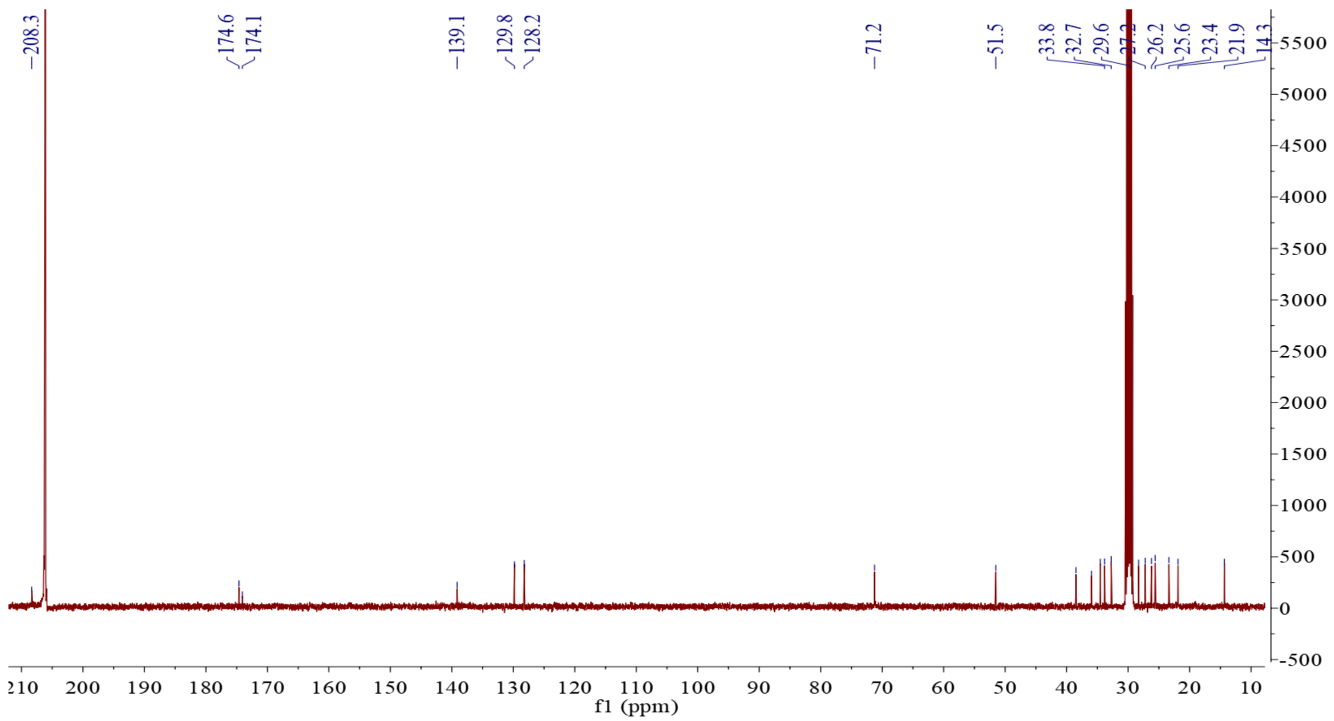

Figure S44. ^{1}H-^{1}H COSY spectrum of **13** in acetone-d_6.

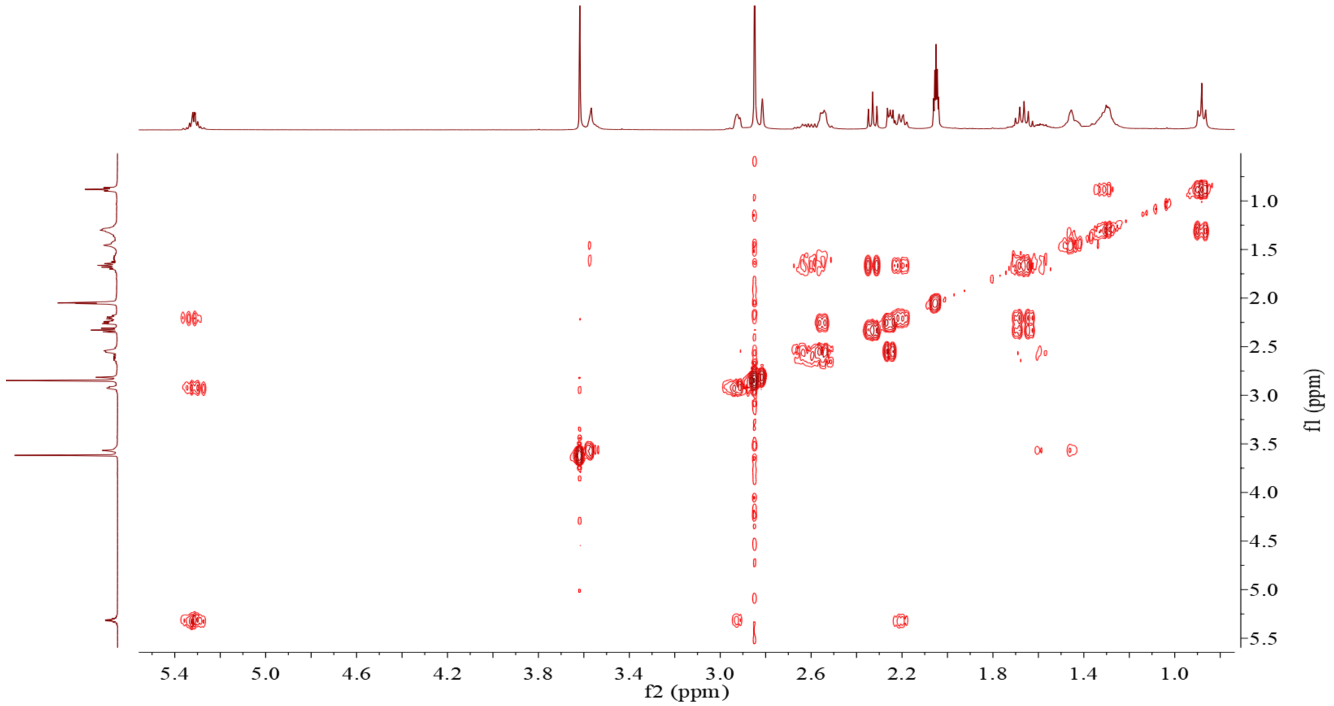

Figure S45. HSQC spectrum of **13** in acetone-d_6.

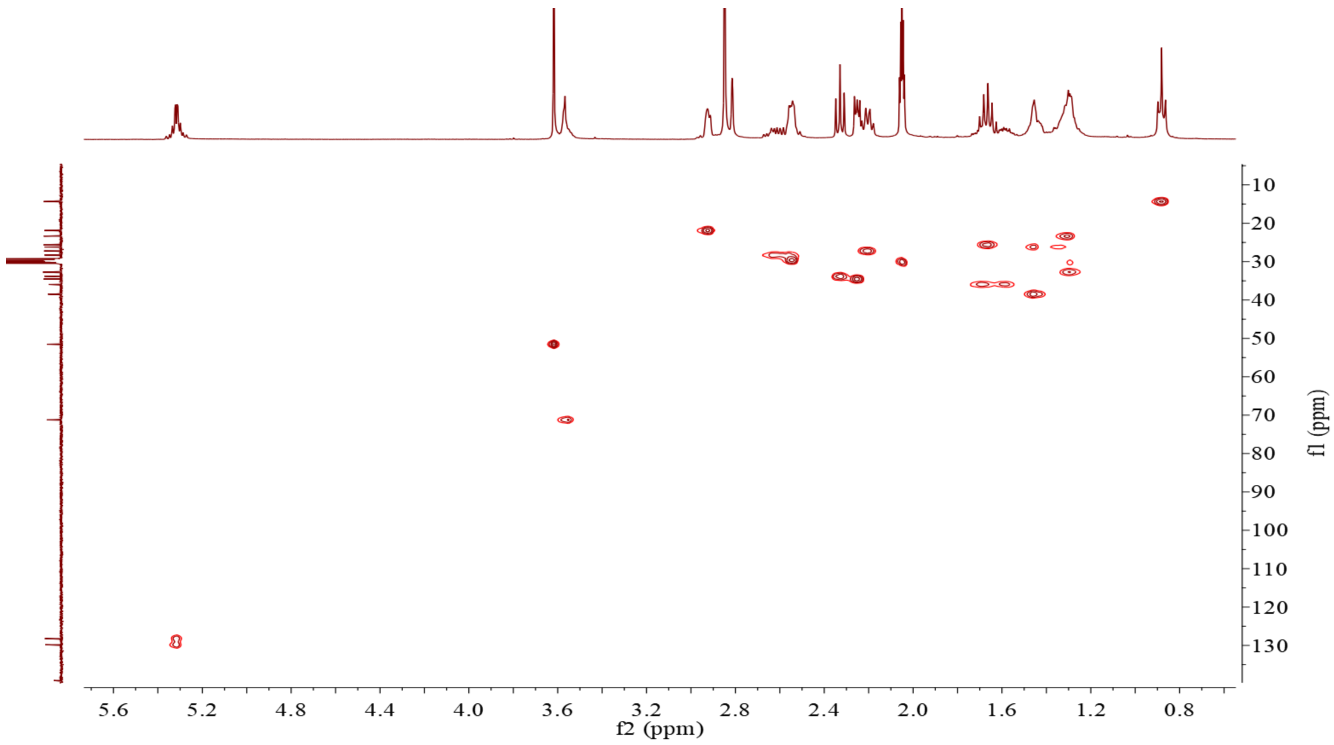

Figure S46. HMBC spectrum of **13** in acetone-d_6

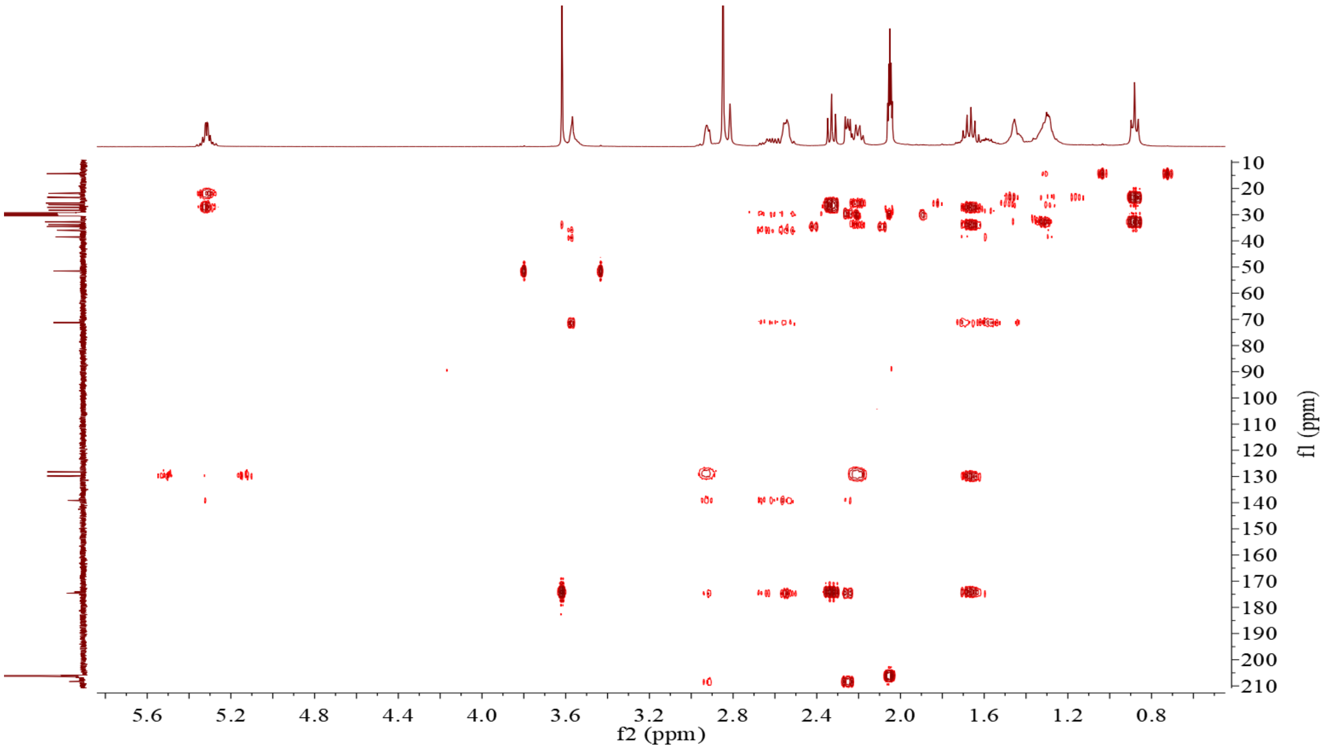

Figure S47. ^{1}H (400 MHz) NMR spectrum of **10a** in pyridine-d_5

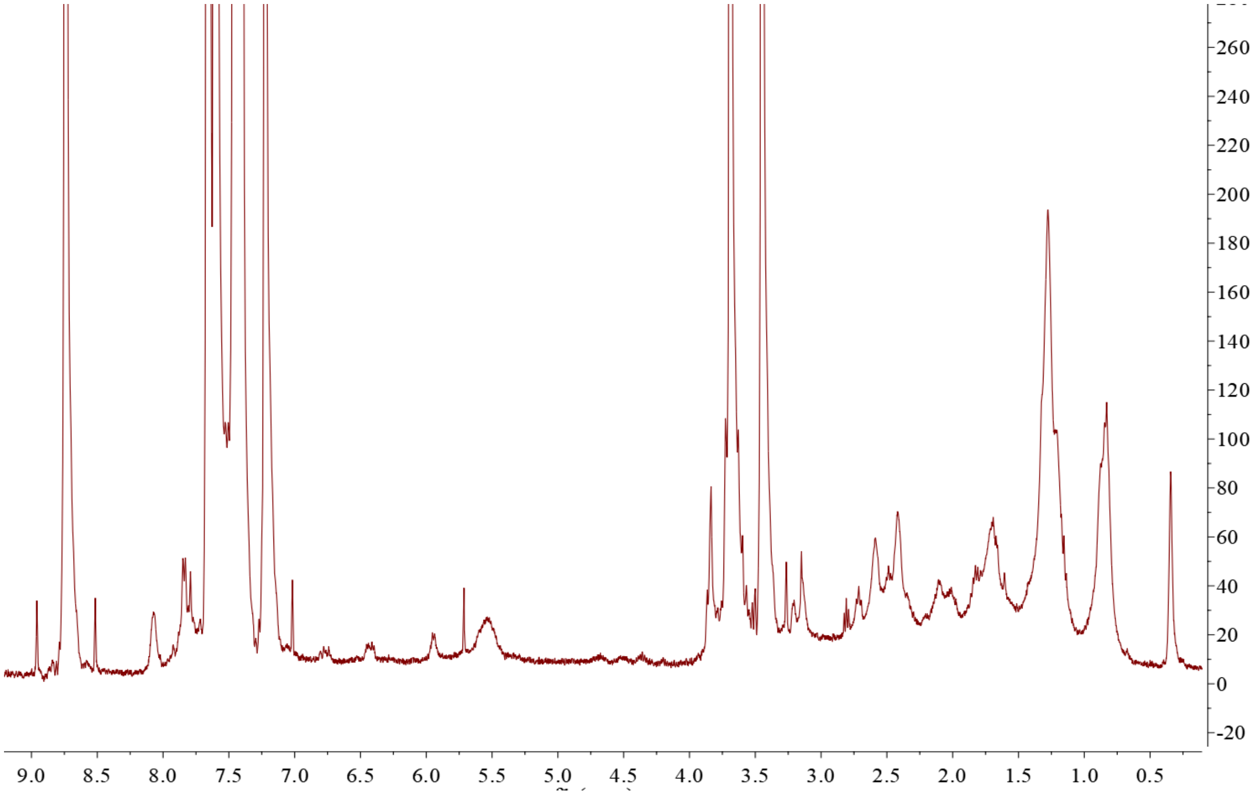

Figure S48. ^{1}H (400 MHz) NMR spectrum of **10b** in pyridine-d_5

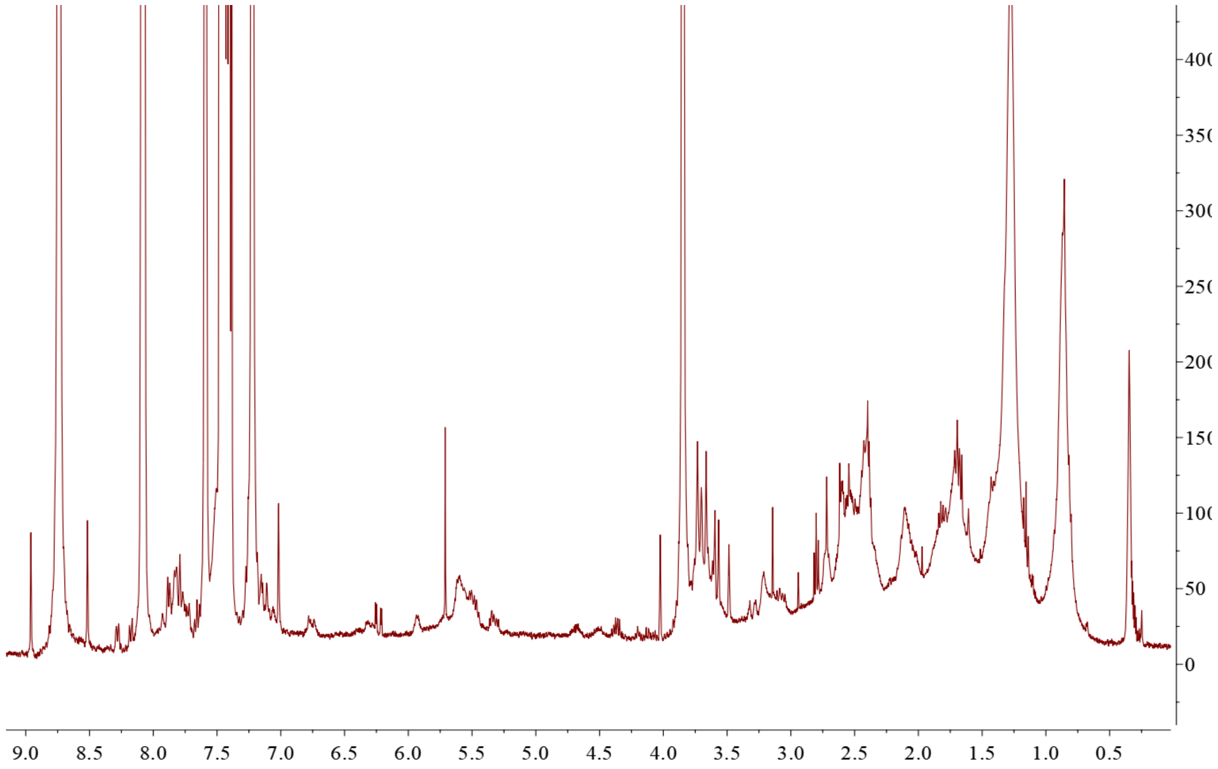

Figure S49. ^{1}H (400 MHz) NMR spectrum of **15a** in pyridine-d_5

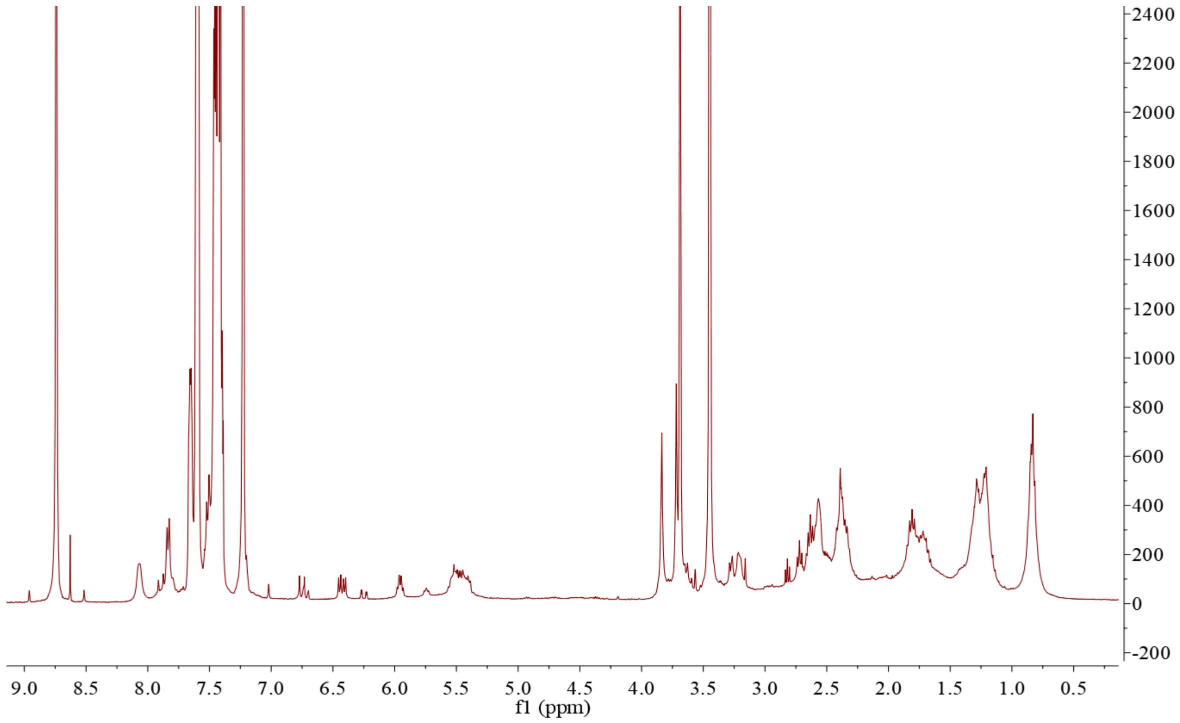

Figure S50. ^{1}H-^{1}H COSY spectrum of **15a** in pyridine-*d*5

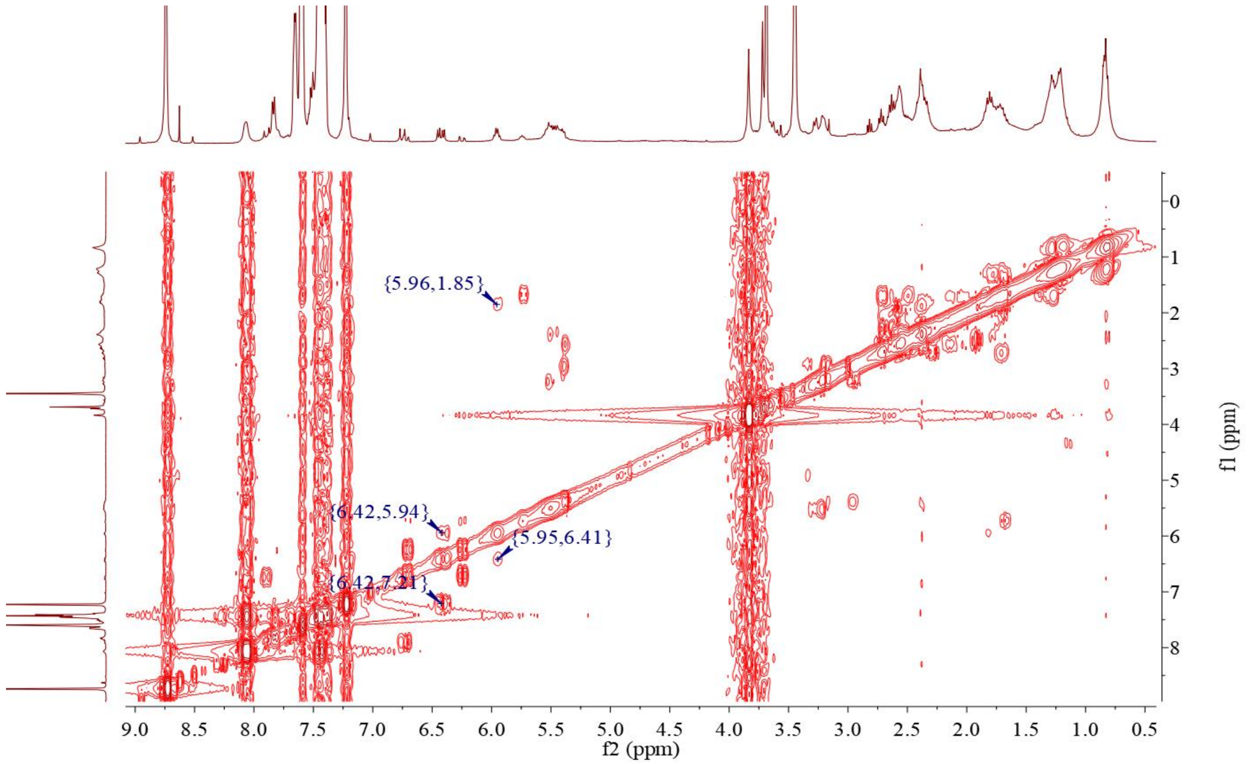

Figure S51. ^{1}H (400 MHz) NMR spectrum of **15b** in pyridine-*d*5

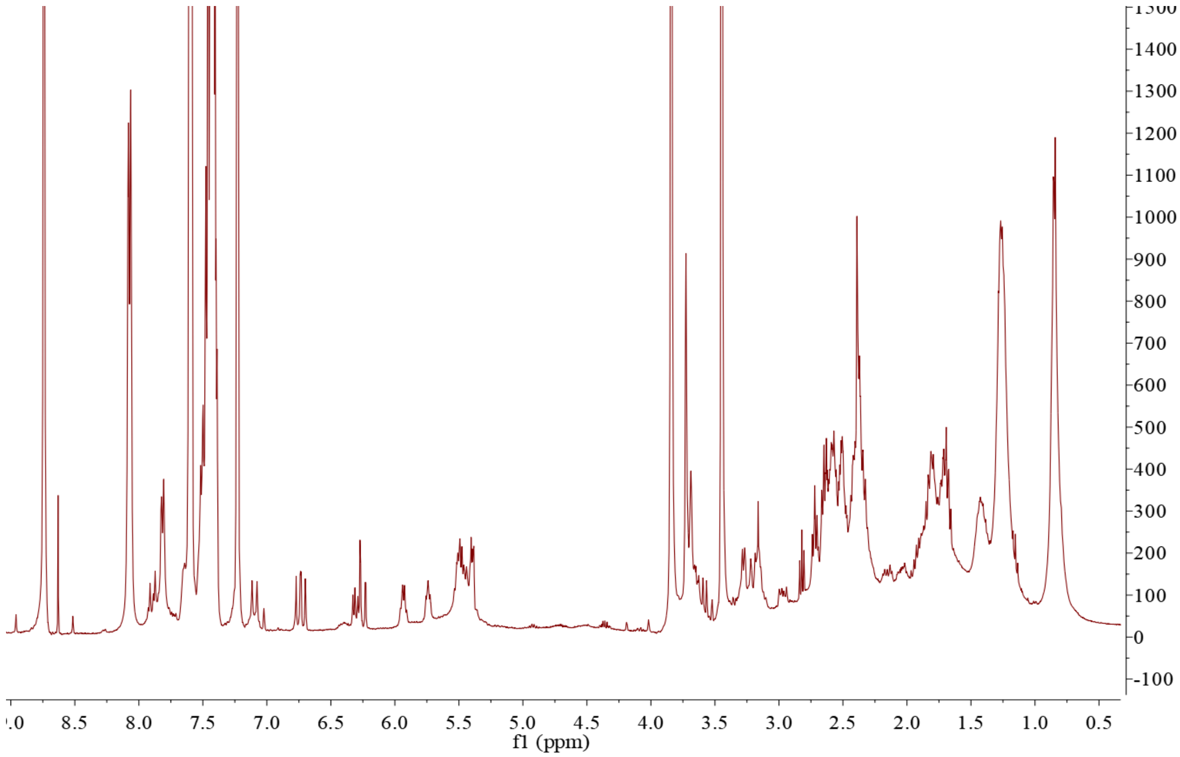

Figure S52. ^{1}H-^{1}H COSY spectrum of **15b** in pyridine-d_5

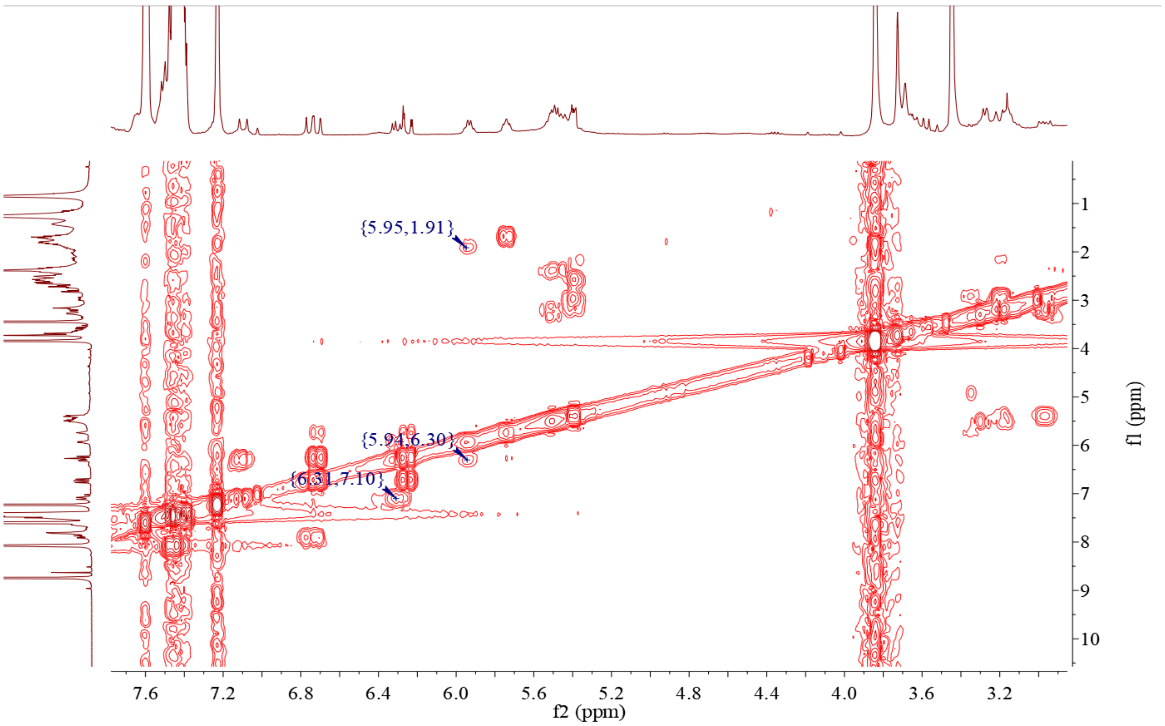

Supplement: Supplementary file 1 [file marinedrugs-17-00481-s001.pdf]
